# The inhibitory effect of hepatic cancer energy metabolism on immune checkpoint therapy: perspectives from single-cell multi-omics analysis

**DOI:** 10.3389/fimmu.2026.1753670

**Published:** 2026-04-13

**Authors:** Xin Li, Ling Tang, Ju Cao, Yao Liu

**Affiliations:** Department of Laboratory Medicine, First Affiliated Hospital of Chongqing Medical University, Chongqing, China

**Keywords:** drug resistance, energy metabolism, hepatocellular carcinoma, immune checkpoint inhibitors, prognosis, pyruvate metabolism, single-cell sequencing

## Abstract

**Objective:**

To investigate metabolic reprogramming—especially pyruvate metabolism—in hepatocellular carcinoma (HCC) before and after immune checkpoint inhibitor (ICI) therapy, construct a metabolism-related prognostic model, and evaluate the therapeutic potential of targeting LDHA.

**Methods:**

Integrated single-cell RNA-seq data (GEO, Mendeley) were analyzed using Seurat, AUCell, pySCENIC, CellChat, and Monocle. A prognostic model was developed from TCGA data by Cox and Lasso regression. Functional validation included *in vitro* CCK8 and Transwell assays in Huh7 cells and *in vivo* xenograft experiments combining the LDHA inhibitor (R)-GNE-140 with a PD-1 antibody.

**Results:**

Post-ICI, HCC cells upregulated pyruvate metabolism genes (LDHA, LDHB, LDHD) but showed decreased glycolysis, lactate buildup, reduced acetylation, and suppressed TCA cycle with AMPK activation. Key transcription factors (MYC, SP5, HLF, SREBF1) were identified. CellChat revealed enhanced SPP1–CD44 and APOA1–ABCA1 signaling. Pseudotime analysis indicated terminal hepatocyte differentiation. The pyruvate metabolism-based signature predicted prognosis effectively. Combination therapy with (R)-GNE-140 and PD-1 blockade inhibited Huh7 proliferation, migration, and xenograft tumor growth without liver or renal toxicity.

**Conclusion:**

ICI therapy induces metabolic remodeling in HCC, marked by pyruvate metabolism dysregulation and lactate accumulation, contributing to resistance. Dual targeting of LDHA and PD-1 enhances antitumor efficacy, and the identified metabolic signature may serve as a prognostic biomarker and therapeutic target.

## 1 Introduction

Hepatocellular carcinoma (HCC), the predominant form of primary liver cancer (accounting for approximately 80%), poses a severe global public health challenge. It ranks as the sixth most common cancer worldwide and the third leading cause of cancer-related deaths (1). Global liver cancer deaths exceed 800,000 annually, and the World Health Organization (WHO) projects that over one million patients will succumb to liver cancer within the next decade. The global incidence of HCC is 9.3 per 100,000 person-years, with a mortality rate of 8.5 per 100,000 person-years (2). This dismal prognosis is largely attributable to late diagnosis, with the majority of patients presenting at intermediate or advanced stages; only about one-third are candidates for potentially curative early-stage treatments such as surgical resection, liver transplantation, or radiofrequency ablation (2). Current therapeutic focus for advanced HCC centers increasingly on combination targeted-immunotherapy, where immunotherapy, particularly immune checkpoint blockade, has become an indispensable component (3).

Currently, atezolizumab (a PD-L1 inhibitor) combined with bevacizumab (a VEGF inhibitor) stands as the first-line recommended regimen for unresectable HCC, demonstrating significant improvements in overall response rate, overall survival, and progression-free survival (4, 5). Furthermore, the dual ICI combination of tremelimumab (a CTLA-4 inhibitor) and durvalumab (a PD-L1 inhibitor) has also gained approval (6). These advances mark a pivotal shift in advanced HCC treatment from single-agent targeted therapy towards multimodal combination immune checkpoint inhibitor (ICI) therapy. However, it should be noted that a significant proportion of patients still derive no benefit from ICI therapy due to the emergence of drug resistance, representing a major challenge limiting the efficacy and durability of ICIs in HCC. Studies indicate that nearly three-quarters of HCC patients develop resistance to immunotherapy: 46.2% exhibit primary resistance (lack of response to treatment), and 28.4% exhibit secondary resistance (disease progression after an initial response) (7). Primary resistance is associated with a significantly worse prognosis, with a median overall survival of only 12.83 months compared to 31.53 months for secondary resistance, underscoring that this high resistance rate directly contributes to poor overall outcomes in HCC patients (7–9).

Emerging evidence implicates tumor metabolism as a key factor in ICI treatment resistance. Cancer cells frequently exhibit abnormally high glycolytic activity, even under aerobic conditions, a phenomenon known as the “Warburg effect” (10). Enhanced glycolysis leads to substantial lactate production. Lactate accumulation acidifies the tumor microenvironment (TME), inhibiting T-cell proliferation, activation, and cytotoxic function, thereby weakening anti-tumor immune responses and promoting immune evasion (11, 12). Lactate can also modulate macrophage polarization towards a pro-tumorigenic M2 phenotype, further suppressing immunotherapy efficacy (13). Pyruvate, the end product of glycolysis, serves as a crucial intermediate for entry into the tricarboxylic acid (TCA) cycle (14). In highly glycolytic cancer cells, a large proportion of pyruvate is converted to lactate by lactate dehydrogenase A (LDHA) rather than entering mitochondrial oxidative phosphorylation (15). High LDHA expression correlates with lower response rates to ICI therapy in HCC patients, suggesting that blocking lactate production might enhance immunotherapy outcomes (15). Nevertheless, two critical scientific questions remain unelucidated: First, whether the process of LDHA-mediated conversion of pyruvate to lactate in hepatocellular carcinoma (HCC) aligns with observations in other tumor types; and second, the precise molecular mechanisms by which pyruvate, lactate, and related metabolites contribute specifically to ICI resistance development in HCC patients.

Addressing these unresolved key scientific questions, this study innovatively integrates multi-omics analytical approaches, including single-cell transcriptomics, to systematically characterize the dynamic remodeling of energy metabolism in HCC before and after ICI therapy. We not only delve into the specificity of the LDHA-mediated pyruvate-lactate metabolic axis in HCC but also employ cell-cell communication analysis and pseudotime trajectory analysis to uncover the specific mechanisms through which metabolic reprogramming influences the immune microenvironment (such as T-cell functional suppression and macrophage polarization), ultimately leading to ICI resistance. Building on these findings, we further leveraged transcriptomic data and employed Lasso regression along with multivariate Cox regression analysis to construct a prognostic signature based on energy metabolism features for HCC. Furthermore, based on the identified key metabolic target (LDHA), this study conducted *in vitro* (Huh7 cells) and *in vivo* (nude mouse xenograft) experiments to validate the anti-tumor efficacy and safety of a combination strategy targeting LDHA ((R)-GNE-140) and PD-1, aiming to provide a direct experimental basis for this therapeutic approach. This model aims to accurately predict patient response to ICI therapy, offering novel potential strategies and a theoretical foundation for overcoming resistance and achieving personalized immunotherapy.

## 2 Materials and methods

### 2.1 Data acquisition

This study aimed to investigate the dynamic alterations in energy metabolism of hepatocellular carcinoma (HCC) before and after immunotherapy. We acquired multiple single-cell RNA sequencing (scRNA-seq) from public databases and supplementary data repositories, integrating them with metabolic pathway gene sets for comprehensive analysis. The GSE151530 dataset was obtained from the Gene Expression Omnibus (GEO) database (https://www.ncbi.nlm.nih.gov/geo/), comprising scRNA-seq data from 46 HCC patients both pre- and post-treatment. From this dataset, we selected paired pre- and post-treatment samples from 7 patients for in-depth analysis: 2 with intrahepatic cholangiocarcinoma (ICC) and 5 with HCC. These patients received combination therapy with durvalumab and tremelimumab (targeting PD-L1 and CTLA-4, respectively). Additionally, the GSE290925 dataset was retrieved from GEO, containing scRNA-seq data from 12 treatment-naïve advanced HCC patients, serving as an independent validation cohort. Finally, a supplementary dataset was acquired from the Mendeley Data platform (https://data.mendeley.com/datasets/skrx2fz79n/1), which included scRNA-seq data from 6 post-treatment HCC samples and their matched adjacent non-tumor tissues. These patients received anti-PD-1 therapy. Furthermore, to systematically analyze energy metabolism pathways, we obtained gene sets corresponding to the following key metabolic pathways (keywords) from the Molecular Signatures Database (MSigDB v7.5.1, https://www.gsea-msigdb.org/gsea/msigdb/): Pyruvate metabolism, Fatty acid metabolism, Glycolysis, Tricarboxylic acid (TCA) cycle, AMPK signaling pathway, and Protein acetylation pathway.

### 2.2 Single-cell data analysis

All acquired scRNA-seq data were processed for standardization and downstream analysis using the Seurat package (v4.3.0) in R. For the GSE151530 dataset, stringent quality control (QC) criteria were applied to remove low-quality cells and doublets: 1) the number of detected genes per cell (nFeature_RNA) > 500; 2) the percentage of mitochondrial genes (percent.mt) < 20%. To mitigate the impact of ambient RNA contamination on cellular expression profiles, filtered data were processed using the decontX package (v1.12.0) in R for free RNA removal. Cells with a contamination score > 0.3 were identified as contaminated and removed (Note: The parameters are default. See **Supplementary Figure 1** for the distribution histogram). To integrate single-cell data from different batches and samples and correct for batch effects, we employed the Harmony package (v0.1.1) in R. During integration, 2000 highly variable genes (HVGs) were selected for analysis. To minimize potential confounding effects from cell cycle phase and mitochondrial gene expression on clustering, the proportion of mitochondrial gene expression and cell cycle scores were regressed out. For dimensionality reduction and clustering, the first 30 principal components (PCs) were used, with a clustering resolution set to 0.5.

Cell type annotation was primarily based on published cell-specific marker genes from the original studies (16, 17). Following annotation, the expression of key immune checkpoint genes CD274 (PD-L1) and CTLA4 across different cell types was visualized. We also analyzed the proportional changes in different cell types pre- and post-treatment. To finely dissect HCC cellular heterogeneity, samples with a high proportion of hepatocytes were selected for subpopulation analysis. For the hepatocyte subpopulation, we again identified 1000 HVGs, performed dimensionality reduction using the first 30 PCs, and clustered the cells at a lower resolution (0.1) to identify finer subpopulations. We compared the proportional changes in hepatocyte subpopulations before and after treatment and identified differentially expressed genes (DEGs) between subpopulations. Differential expression analysis was performed using the Wilcoxon rank-sum test, with P-values adjusted using the Bonferroni correction. Genes with an adjusted P-value < 0.05 and |log2(Fold Change)| > 0.25 were considered significantly differentially expressed.

For the identified highly expressed DEGs, Gene Ontology (GO) and Kyoto Encyclopedia of Genes and Genomes (KEGG) pathway enrichment analyses were conducted using the clusterProfiler package (v4.4.4) in R to elucidate the functional characteristics of the differential subpopulations, with a specific focus on key pathways related to pyruvate metabolism. We further analyzed the expression patterns of genes within these key pathways and inferred potential sources of pyruvate based on gene expression.

### 2.3 Lactylation and acetylation regulation analysis

To assess the activity of the TCA cycle and protein acetylation pathways, we calculated the activity score for each cell using the corresponding gene sets via the AUCell package (v1.18.0) in R (18). We further analyzed the expression patterns of genes encoding protein acetylation “writers” and “erasers” across different hepatocyte subpopulations to reveal the role of acetylation modifications in HCC cell metabolic reprogramming.

### 2.4 Transcription factor analysis

Transcription factor (TF) activity analysis within hepatocytes was performed using the pySCENIC workflow (v0.11.0) to identify TFs specifically enriched in different hepatocyte clusters (19). We identified TFs exhibiting high expression and specific regulatory activity within each cluster. High-specificity TF regulatory networks were obtained by intersecting these TFs with highly expressed genes in each cluster. Additionally, we utilized the Cistrome DB database (http://cistrome.org/db/#/) to validate the potential regulatory roles of the top 5 most significantly differential TFs on the key metabolic gene LDHA.

### 2.5 Cell-cell communication analysis

Cell-cell communication analysis was performed on pre- and post-treatment single-cell data using the CellChat package (v1.6.1) in R. We compared differences in cell-cell communication patterns within the HCC microenvironment before and after treatment, focusing on ligand-receptor interactions between T cells, hepatocytes, and other immune or stromal cells, to reveal how immunotherapy remodels the cellular communication network.

### 2.6 Global metabolic pathway activity assessment

To comprehensively evaluate the energy metabolic state in HCC cells, we again employed the AUCell package to calculate activity scores for the Pyruvate metabolism, Fatty acid metabolism, Glycolysis, TCA cycle, AMPK signaling pathway, and Protein acetylation pathway across all cell types. The expression distribution of these pathways within different cell clusters was visualized.

### 2.7 External dataset validation

To validate the generalizability of our discovered metabolic signatures across different HCC cohorts, we analyzed external scRNA-seq datasets (GSE290925 and the post-treatment scRNA-seq data from the Mendeley dataset). Processing of the GSE290925 dataset was similar to GSE151530, with specific parameters: nFeature_RNA > 500, percent.mt < 25%, nCount_RNA > 500. Ambient RNA removal was also performed using decontX (contamination score < 0.3). During batch effect correction, the number of HVGs was set to 3000, mitochondrial and cell cycle scores were regressed out, dimensionality reduction and clustering used the first 30 PCs, and resolution was set to 0.5. The cell cycle distribution was evaluated by comparing the sample distributions before and after PCA, as detailed in **Supplementary Figure 2**.

The post-treatment scRNA-seq data from the Mendeley dataset had undergone prior QC and filtering; therefore, it was used directly for subsequent analysis without additional filtering. We merged hepatocyte data from pre-treatment (GSE151530) and post-treatment (Mendeley dataset) cohorts to analyze changes in cell proportions. Subsequently, activity scores for Pyruvate metabolism, Fatty acid metabolism, Glycolysis, TCA cycle, AMPK signaling pathway, and Protein acetylation pathway were calculated using the AUCell package and assessed within the merged hepatocyte dataset. Furthermore, we analyzed the expression of pyruvate-related genes and key TF genes in these validation cohorts.

### 2.8 Pseudotime trajectory analysis

To uncover potential differentiation trajectories of HCC cells during treatment, pseudotime trajectory analysis was performed on hepatocytes from the GSE151530 dataset using the Monocle package (v2.24.0) in R. We tracked the expression distribution of key metabolic genes (LDHA, LDHB, LDHD) and two core TFs along the pseudotime trajectory to infer their temporal regulation in HCC cell metabolic reprogramming.

### 2.9 TCGA prognostic, clinical, immune infiltration analysis and nomogram construction

To further validate the clinical prognostic value of metabolism-related genes, transcriptomic data and corresponding clinical information for HCC patients (TCGA-LIHC) were downloaded from The Cancer Genome Atlas (TCGA) database. Genes related to pyruvate metabolism and their expression levels were extracted from the gene expression matrix. Univariate Cox proportional hazards regression survival analysis was performed on pyruvate metabolism-related genes using the survival package (v3.5-7) in R, selecting genes with P < 0.05 as significantly associated with prognosis. Subsequently, a prognostic model based on Least Absolute Shrinkage and Selection Operator (Lasso) regression was constructed using the glmnet package (v4.1-8) in R. The dataset was randomly divided into a training cohort (70%) and a testing cohort (30%). Ten-fold cross-validation was performed within the training cohort to optimize model parameters.

Patients were stratified into high-risk and low-risk groups based on the model risk score, and survival differences between groups were compared. We also analyzed the correlation between the model risk score and clinical characteristics (e.g., age, sex, tumor TNM stage, pathological stage). The CIBERSORT algorithm (v1.03) was used to estimate the proportions of different immune cell subpopulations infiltrating the tumor in the TCGA-LIHC cohort. Differences in immune cell infiltration proportions between high-risk and low-risk groups were compared to explore the association between metabolic signatures and the tumor immune microenvironment. Univariate and multivariate Cox regression analyses were performed to evaluate the independent prognostic value of the model risk score and key clinical data. Finally, a nomogram was constructed using the rms package (v6.6-0) in R, integrating the independent prognostic factors to provide a visual, individualized prognostic prediction tool for clinical practice.

### 2.10 Cell culture and reagents

The mouse hepatocellular carcinoma cell line Huh7 was obtained from the American Type Culture Collection (ATCC, Manassas, VA, USA). Cells were cultured in high-glucose Dulbecco’s Modified Eagle Medium (DMEM) (Gibco, USA) supplemented with 10% Fetal Bovine Serum (FBS) (Gibco, USA) and 1% Penicillin-Streptomycin solution (Solarbio, China). Cells were maintained in a humidified incubator at 37 °C with 5% CO2. The PD-1 antibody (InVivoMab anti-mouse PD-1, Clone RMP1-14) was purchased from Bio X Cell (USA). The LDHA small molecule inhibitor ((R)-GNE-140) was purchased from Selleck Chemicals (USA) and dissolved in DMSO for *in vitro* use.

### 2.11 *In vitro* assays CCK8 assay

Huh7 cells were seeded into 96-well plates at a density of 5×10^3^ cells/well and allowed to adhere overnight. Cells were then divided into four groups: Control (DMSO vehicle), Anti-PD-1 (10 µg/mL), LDHA Inhibitor ((R)-GNE-140, 10 µM), and Anti-PD-1+LDHA Inhibitor combination. Cell viability was measured at 0h, 24h, 48h, and 72h. At each time point, 10 µL of CCK8 reagent (Dojindo, Japan) was added to each well and incubated for 2 hours at 37 °C. The absorbance was measured at 450 nm using a microplate reader (Thermo Fisher Scientific, Varioskan LUX).

### 2.12 Transwell migration assay

Cell migration was assessed using Transwell chambers (8.0 µm pore size, Corning, USA). 5×10^4^ Huh7 cells in 200 µL of serum-free DMEM (containing the respective treatments: Control, Anti-PD-1, LDHA Inhibitor, or combination) were seeded into the upper chamber. The lower chamber was filled with 600 µL of DMEM containing 10% FBS as a chemoattractant, also including the respective treatments. After 24 hours of incubation, cells that did not migrate were removed from the upper surface of the membrane with a cotton swab. Cells that had migrated to the lower surface were fixed with 4% paraformaldehyde for 20 minutes and stained with 0.1% crystal violet for 15 minutes. Migrated cells were imaged and counted in five random fields under an inverted microscope.

### 2.13 Animal xenograft model and sample analysis

All animal procedures were approved by the Institutional Animal Care and Use Committee of Laboratory Animal Center of Chongqing Medical University (IACUC-CQMU). Six-week-old male BALB/c nude mice (n=6 per group) were subcutaneously injected in the right flank with 5×10^6^ Huh7 cells. When tumors reached approximately 100 mm³, mice were randomized into four groups: Control (vehicle), Anti-PD-1 (10 mg/kg), LDHA Inhibitor ((R)-GNE-140, 25 mg/kg), and Anti-PD-1+LDHA Inhibitor combination. Treatments were administered via intraperitoneal (i.p.) injection three times weekly for three weeks. Tumor volume (V=(length×width^2^)/2) and body weight were monitored every 3 days. On day 18, mice were euthanized, and tumors and orbital blood were collected. Tumors were weighed, fixed in 10% formalin, paraffin-embedded, and sectioned for Hematoxylin and Eosin (HE) staining. Tumor necrosis was assessed by pathological scoring. Serum was isolated from orbital blood and analyzed for liver (ALT, AST) and kidney (BUN, Cre) function markers.

### 2.14 Statistical analysis

All *in vitro* and *in vivo* experimental data are presented as mean ± standard deviation (SD). Statistical analyses were performed using GraphPad Prism 9.0 (GraphPad Software, USA). Differences between two groups were assessed using a two-tailed Student’s t-test. Differences among multiple groups were assessed using one-way ANOVA (for endpoint data like Transwell counts or final tumor weight) or two-way ANOVA (for time-course data like CCK8 or tumor growth curves), followed by Tukey’s *post-hoc* test. A P-value < 0.05 was considered statistically significant. Significance levels are denoted as *p < 0.05; **p < 0.01; ***p < 0.001; **** p < 0.0001.

## 3 Result

### 3.1 Characterization of the HCC TME and ICI-driven reprogramming of pyruvate metabolisms

**Figure 1:**
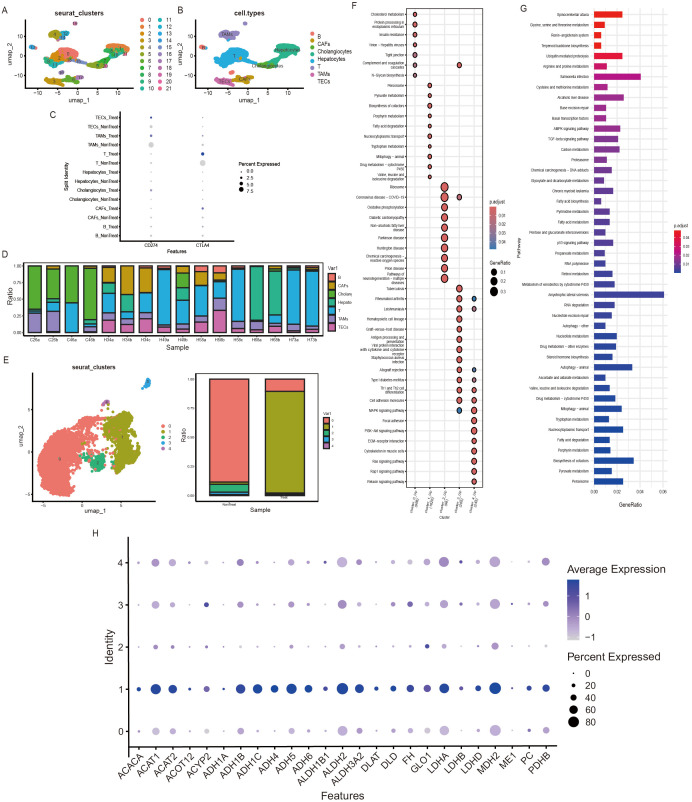
Single-cell analysis of HCC TME and hepatocyte metabolic reprogramming. **(A)** UMAP dimensionality reduction plot showing 22 initial cell clusters. **(B)** Annotation of 7 major cell populations based on established marker genes. **(C)** Expression patterns of CD274 (PD-L1) and CTLA4 in various cell types before and after immunotherapy. **(D)** Cellular composition across samples. Sample H68 exhibits the highest proportion of Hepatocytes. **(E)** UMAP plot of hepatocyte subclustering analysis based on metabolic-related gene expression profiles, identifying 5 subpopulations (clusters 0-4). Cluster 1 is significantly enriched in post-treatment samples. **(F)** KEGG pathway enrichment analysis reveals functional clustering of hepatocyte subpopulations (clusters 0-4). **(G)** Hierarchical clustering of significantly enriched pathways within these clusters. **(H)** Expression patterns of core genes within the Pyruvate metabolism pathway across the five hepatocyte subpopulations. Genes in this pathway exhibit significantly elevated expression in Cluster 1.

We first acquired the single-cell transcriptomic dataset (ID: GSE151530) from the GEO database, comprising hepatocellular carcinoma (HCC) samples from 46 patients treated with dual immune checkpoint (PD-L1/CTLA-4) inhibitor (Durvalumab + Tremelimumab). To dynamically dissect the remodeling effects of ICI therapy on energy metabolism within the HCC tumor microenvironment (TME), we selected 7 patients with high-quality paired pre- and post-treatment samples. Integrated single-cell analysis was then performed, followed by dimensionality reduction using UMAP and unsupervised clustering, which identified 22 distinct cell populations (**Figure 1A**). Subsequently, cell clusters were annotated using established cell type marker genes (17, 20). This annotation defined 7 major cell populations (**Figure 1B**): T cells, B cells, Cancer-associated fibroblasts (CAFs), Tumor-associated macrophages (TAMs), Tumor endothelial cells (TECs), Liver progenitor cells (HPCs), and Cholangiocytes.

To validate the dataset’s reliability in reflecting therapeutic responses, we analyzed the expression changes of key immune checkpoint molecules CD274 (PD-L1) and CTLA-4 before and after treatment. The results indicated significant reductions in CD274 and CTLA-4 expression levels within T-cell subsets post-treatment (**Figure 1C**, **Supplementary Figure 3**). This phenomenon is consistent with the mechanisms of Durvalumab (targeting PD-L1) and Tremelimumab (targeting CTLA-4), demonstrating that dual-targeted immunotherapy, which blocks PD-1/PD-L1 and CTLA-4, operates at various stages of T-cell activation, thereby synergistically enhancing immune responses against cancer cells. This also confirms the anticipated targeted therapeutic effects of the therapy.

Further analysis of cellular composition revealed significant variation in the proportion of hepatocytes among different samples. Notably, sample H68 showed the highest proportion of hepatocytes (**Figure 1D**). As research shows, hepatocellular carcinoma primarily originates from hepatocytes, while benign lesions arise from hepatic progenitor cells (DOI: 10.1016/j.celrep.2017.03.059). Therefore, this study selected hepatocyte populations from H68 samples to conduct in-depth metabolic analysis focusing on energy metabolism, aiming to obtain more representative metabolic characteristics of malignant HCC cells.

To investigate the metabolic effects of ICI therapy on HCC hepatocytes, we analyzed hepatocytes from a representative patient (H68) before and after treatment. UMAP-based clustering of metabolism-related gene expression identified five hepatocyte subpopulations (clusters 0–4) with distinct metabolic profiles (**Figure 1E**). Cluster 1 was notably enriched after treatment, suggesting its association with therapeutic response or metabolic adaptation.

KEGG enrichment analysis showed that cluster 1 was specifically enriched in core metabolic pathways, including Peroxisome, Pyruvate metabolism, Biosynthesis of cofactors, Porphyrin metabolism, Fatty acid degradation, Nucleocytoplasmic transport, Tryptophan metabolism, Mitophagy (autophagic clearance of mitochondria), Drug metabolism - cytochrome P450, and Valine, leucine and isoleucine degradation (**Figure 1F**). Hierarchical clustering further highlighted fatty acid metabolism, AMPK signaling, and DNA repair pathways as functionally connected networks (**Figure 1G**).

Based on the functional clustering analysis (**Figure 1G**), which revealed the central role of the pyruvate metabolism pathway within the hepatocyte subpopulation (cluster 1) enriched after ICI therapy, and considering its established pivotal role in tumor progression and drug resistance (21), we hypothesized that pyruvate metabolic reprogramming may underlie adaptive resistance to ICI therapy. Analysis of key pathway genes revealed that PDHB, DLAT, and DLD - enzymes converting lactate to pyruvate—were highly expressed in cluster 1, indicating enhanced pyruvate production. Additionally, PC and alcohol dehydrogenase genes showed elevated expression, supporting active TCA cycle replenishment and P450-related metabolic activity (22, 23) (**Figure 1H**).

### 3.2 Disrupted lactate efflux and TF-mediated pyruvate metabolic reprogramming shape ICI-resistant hepatocyte phenotypes

**Figure 2:**
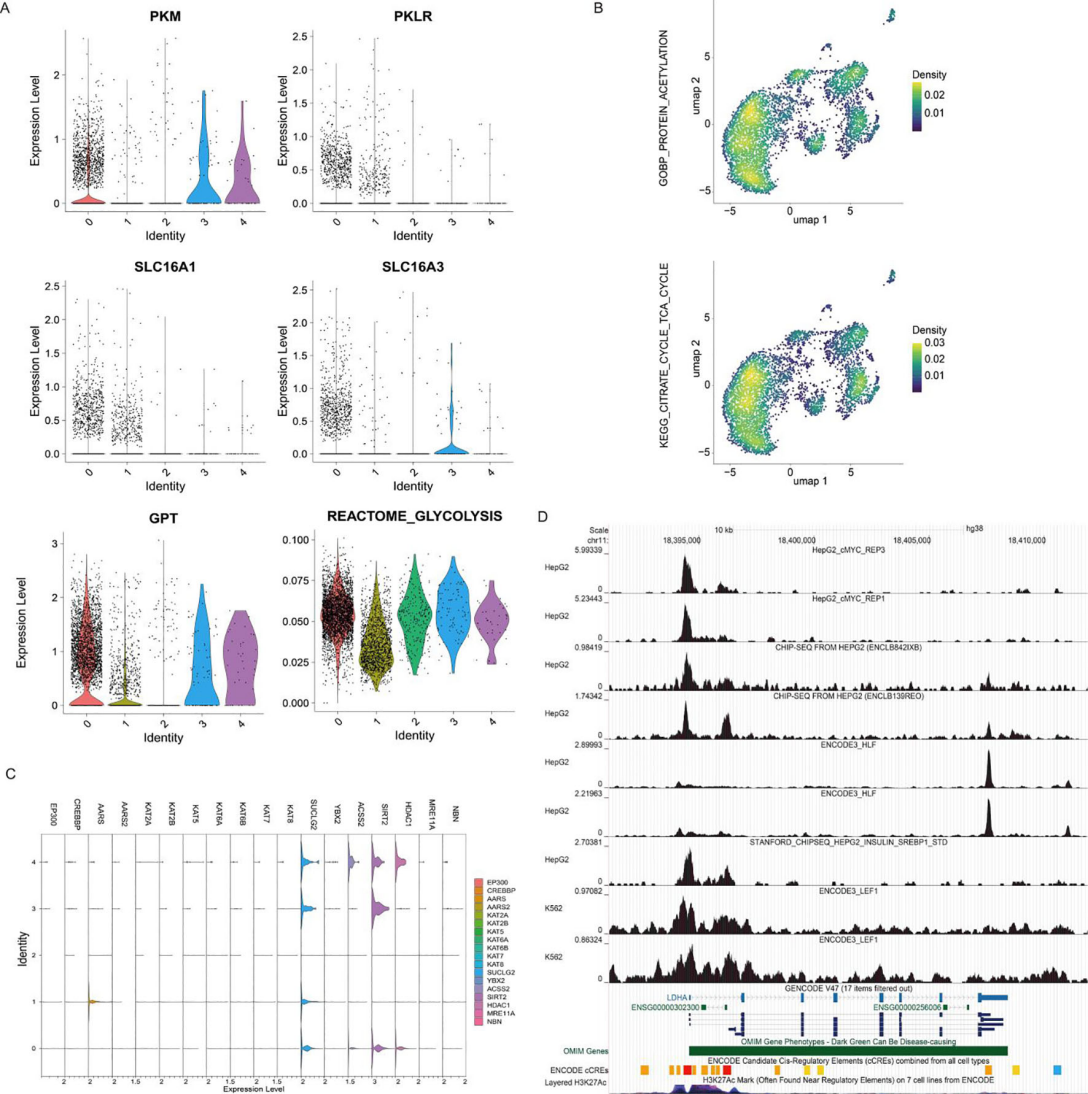
Expression, distribution, and transcription factor analysis of metabolism-related genes. **(A)** Expression of lactate source-related genes and glycolytic pathway activity score. **(B)** Distribution of protein acetylation and TCA cycle activity scores; both protein acetylation levels and TCA cycle scores are significantly reduced in Cluster 1. **(C)** Expression of lactylation ‘writer’ and ‘eraser’ genes (x-axis: expression level; y-axis: different cell clusters; colors represent different genes). **(D)** Cistrome DB peak plots demonstrating transcription factor binding. Lactate Accumulation and MYC-Dependent Pyruvate Metabolic Reprogramming in Post-ICI Hepatocytes.

To investigate the underlying reasons for the high expression of key pyruvate metabolism genes (such as LDHA) in the post-ICI enriched hepatocyte subpopulation and its metabolic context, we further analyzed the expression patterns of genes related to upstream pyruvate sources (primarily glycolysis) and lactate metabolism (**Figure 2A**). Lactate influx genes (PKM, SLC16A3) were largely unchanged, whereas efflux-associated genes (PKLR, GPT, SLC16A1) were markedly downregulated, indicating impaired lactate export. Cluster 1 exhibited the lowest glycolytic activity, suggesting reduced glycolytic flux and *de novo* pyruvate synthesis, coupled with intracellular lactate accumulation (“lactate entrapment”). Given that lactate serves as a primary precursor for pyruvate and supports chemoresistance in HCC (11), these results implicate the pyruvate–lactate metabolic axis in ICI resistance.

Moreover, Sustained expression of SLC16A1/MCT1 suggests ongoing lactate uptake from the microenvironment, exacerbating intracellular accumulation. Excess lactate can drive intracellular acidification and immunosuppression via mTOR and HIF1α signaling (11), indicating that “lactate entrapment” reflects a metabolic adaptation enabling hepatocytes to survive under ICI pressure. Building on the distinct lactate metabolism profile of hepatocytes—marked by high LDHA expression, reduced glycolysis, and impaired lactate efflux leading to intracellular accumulation—we explored its effects on downstream post-translational modifications (PTMs). We focused on lactylation and acetylation, two metabolically linked PTMs competing for CoA-derived substrates that can influence each other’s activity and modulate gene expression and metabolic adaptation. We hypothesized that disrupted lactate metabolism may alter the balance between these modifications, contributing to resistance-associated phenotypes.

Pathway scoring revealed significantly lower global protein acetylation levels in hepatocytes compared to other subpopulations (**Figure 2B**). Concurrently, enrichment of the AMPK signaling pathway suggested an energy-deficient state, indicating that acetyl-CoA was likely diverted toward the TCA cycle. Consistent with this, TCA cycle activity was markedly reduced in cluster 1 (**Figure 2B**, **Supplementary Figure 4**). Using the GSVA scoring method, we still observed the aforementioned pattern: both clusters 1 and 2 exhibited relatively low protein acetylation and tricarboxylic acid (TCA) cycle scores (**Supplementary Figure 5**), further supporting the robustness of our results.

Considering that intracellular lactate accumulation (a hallmark of cluster 1) is a key driver of protein lactylation, we further analyzed the expression profiles of key enzymes regulating lactylation and acetylation modifications across hepatocyte subpopulations (**Figure 2C**). Based on literature (24), we examined known or potential lactylation “writers” (e.g., EP300/p300, KAT2B/PCAF, KAT6B/MOZ, possessing lactyltransferase activity) and “erasers” (delactylation enzymes). Analysis showed that among lactylation writers, EP300, AARS, KAT2B, and KAT6B exhibited higher expression in cluster 1. While Lactylation erasers were lower in cluster 1. The writers and erasers for acetylation and lactylation largely overlapped. Compared to pre-treatment states, this pattern suggests reduced overall PTM dynamics (both writing and erasing) in post-treatment hepatocytes, although the selective upregulation of specific writers (EP300, AARS, KAT2B, KAT6B) might represent an adaptive response to drug pressure. We also examined the expression of key DNA repair genes MRE11A and NBN, finding higher expression in cluster 1 (**Figure 2C**). This raises the possibility that acetylation and lactylation modifications could enhance the DNA repair function of these proteins, potentially aiding resistance to drug-induced stress.

To elucidate the upstream regulatory mechanisms underlying the elevated expression of pyruvate metabolism genes (e.g., LDHA) and the associated metabolic reprogramming in the ICI-enriched hepatocyte subpopulation (cluster 1), we performed transcription factor (TF) regulatory network analysis. Considering that TFs orchestrate cellular adaptation to metabolic and therapeutic stress, we hypothesized that the distinct metabolic phenotype of cluster 1 is driven by specific TF activity.

Using the pySCENIC algorithm, we reconstructed single-cell regulatory networks for HCC (HepG2) and reference (K562) cells. Comparative analysis identified 15 TFs with significantly increased activity in cluster 1, including MYC, SP5, HLF, SREBF1, LEF1, HMGN3, NFE2L2 (NRF2), KLF7, BCLAF1, MSC, TGIF1, ATF4, CEBPB, MEIS2, and NNT.Focusing on TFs with established roles in metabolic regulation (MYC, SP5, HLF, SREBF1, LEF1), we interrogated ChIP-Seq data from HepG2 cells using CistromeDB. As depicted in **Figure 2D**, the binding sites of key transcription factors (TFs), such as MYC, HLF, and LEF1—were significantly enriched within the promoter and enhancer regions of core pyruvate metabolism genes (such as LDHA, LDHB, PKM, etc.), supporting their role in the transcriptional regulation of this pathway within cluster 1.

### 3.3 Remodeling of metabolic and intercellular communication networks within the tumor microenvironment after ICI therapy

Recognizing the tumor microenvironment (TME) as a highly interconnected ecosystem, metabolic alterations in tumor cells can profoundly impact the functional state of neighboring immune cells (e.g., T cells, TAMs) via secreted factors or surface molecules, thereby shaping an immunosuppressive microenvironment and mediating resistance. To test this hypothesis and systematically analyze the remodeling of intercellular communication networks within the TME by ICI therapy, we conducted comprehensive cell-cell communication analysis on paired pre- and post-treatment samples using CellPhoneDB.

**Figure 3:**
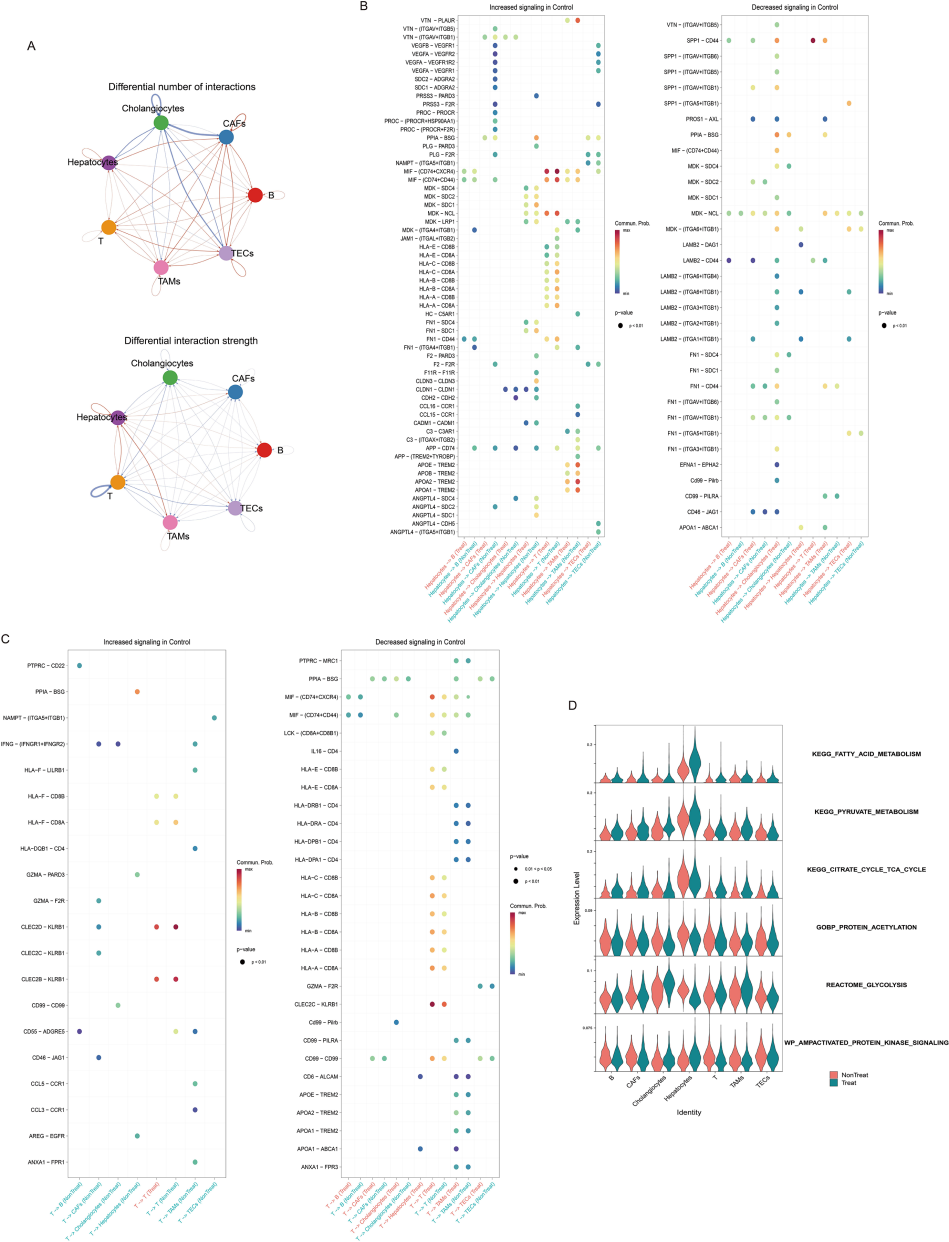
Cell-cell communication analysis and metabolic validation. **(A)** Differential cell-cell communication network weights. **(B)** Differentially significant ligand-receptor interactions originating from hepatocytes. **(C)** Differentially significant ligand-receptor interactions originating from T cells. **(D)** Metabolic pathway activity scores across all cell types.

Analysis of pre- and post-treatment single-cell data revealed a significant increase in the overall interaction weight post-treatment, while the number of significant ligand-receptor pairs remained relatively unchanged (**Figure 3A**). Within the post-treatment group, specific interactions were prominent: SPP1-CD44 interactions showed high probability between hepatocytes and T cells, and between hepatocytes and TAMs. Conversely, interactions along the APOA1-ABCA1 axis exhibited high probability between T cells and hepatocytes, and between TAMs and hepatocytes (**Figures 3B, C**).

To delineate the overall role of metabolism in HCC before and after therapy, we performed metabolic pathway activity scoring across all cell clusters. Analysis revealed distinct metabolic shifts post-treatment (**Figure 3D**, **Supplementary Figure 6**). Fatty acid metabolism scores exhibited an increasing trend in all cell types post-treatment, with this increase being particularly pronounced in hepatocytes. Similarly, pyruvate metabolism scores were elevated in the post-treatment group across cell types. In contrast, TCA cycle scores displayed a cell-type-specific pattern: they decreased in hepatocytes and cholangiocytes but increased in other cell types. Protein acetylation scores followed a similar pattern, decreasing in hepatocytes and cholangiocytes. Conversely, AMPK signaling pathway scores increased in hepatocytes and cholangiocytes. Glycolysis scores decreased in hepatocytes but increased in cholangiocytes post-treatment (**Figure 3D**). This differential utilization of glycolysis between hepatocytes (HCC) and cholangiocytes (potentially ICC) may underlie their disparate responses to PD-L1/CTLA-4 therapy.

### 3.4 External dataset validation

**Figure 4:**
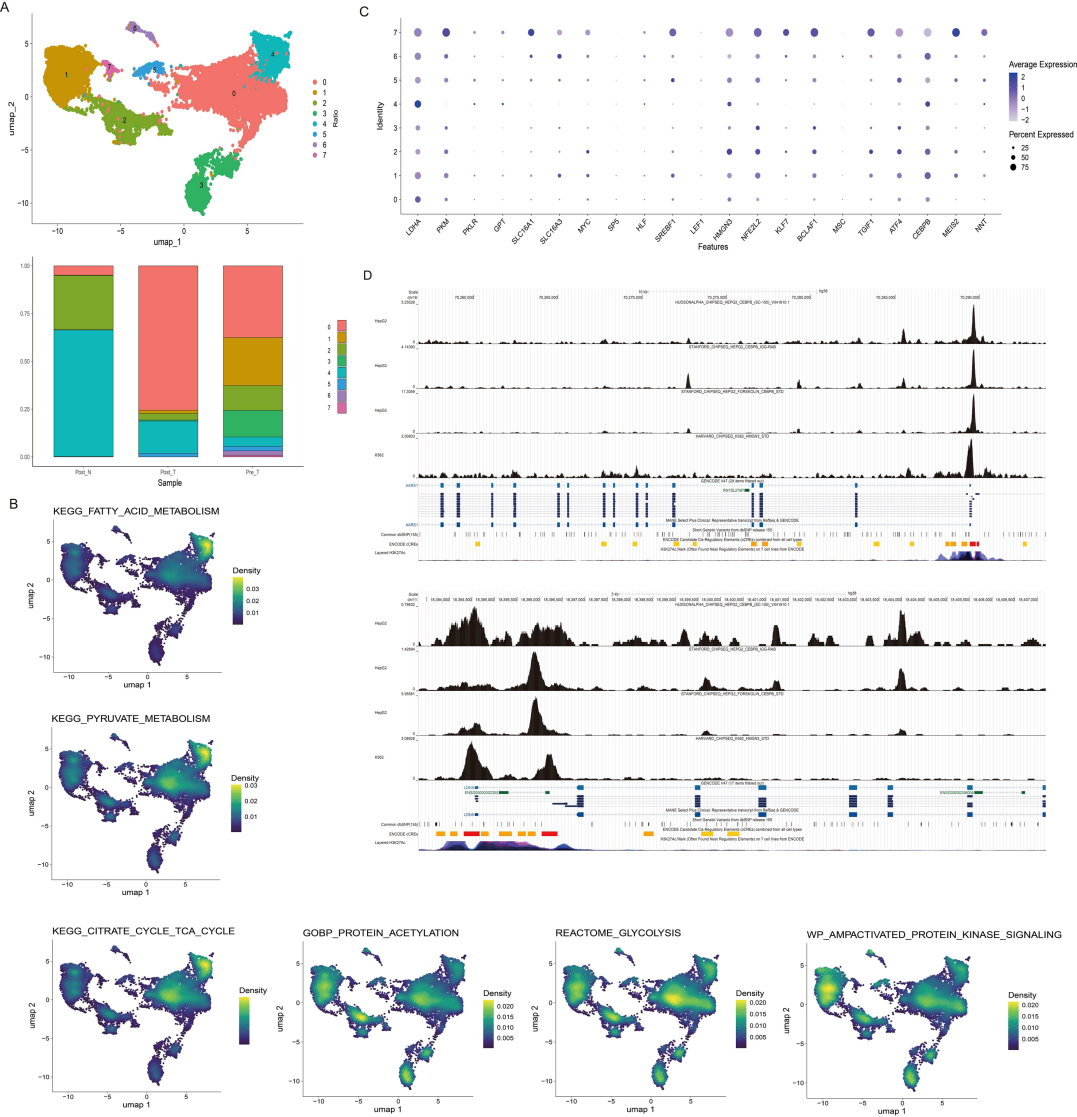
External dataset clustering and functional validation. **(A)** Clustering validation using merged external datasets (pre-treatment + anti-PD-1 post-treatment hepatocytes). **(B)** Aggregate pathway activity scores from all clusters. **(C)** Bubble charts showing specific gene expression (Pyruvate source (mainly glycolysis) and lactic acid metabolism-related genes) analysis for each cluster. **(D)** The top panel shows the peak profiles of transcription factors HMGN3 and CEBPB at the AARS genomic locus in the Cistrome DB database, while the bottom panel displays their profiles at the LDHA locus.

To validate our findings in an independent cohort, we merged hepatocyte data from pre-treatment samples (GSE151530 dataset) with post-treatment hepatocytes from the Mendeley dataset (treated with anti-PD-1 therapy), resulting in 8 distinct clusters. Post-treatment hepatocytes were predominantly localized in clusters 0 and 4, while adjacent non-tumor hepatocytes were mainly found in clusters 2 and 4 (**Figure 4A**). Pathway activity scoring within these merged clusters showed that pyruvate metabolism, fatty acid metabolism, and TCA cycle scores were all highest in cluster 4. Glycolysis scores were highest in cluster 0. Protein acetylation scores peaked in cluster 2, and AMPK signaling pathway scores were highest in cluster 1 (**Figure 4B**). Examining specific genes, expression of the pyruvate-related gene LDHA was elevated in cluster 4, accompanied by reduced PKM expression (**Figure 4C**). Furthermore, the transcription factors HMGN3 and CEBPB showed high expression in cluster 4 (**Figure 4C**). Validation using Cistrome DB confirmed the presence of significant binding peaks for both HMGN3 and CEBPB at the genomic loci of LDHA and AARS (**Figure 4D**, **Supplementary Table 3**). Collectively, these results substantiate that anti-PD-1 therapy induces elevated pyruvate metabolism, fatty acid metabolism, TCA cycle activity, and glycolysis in HCC hepatocytes, alongside lactate accumulation, contributing to the development of drug resistance.

### 3.5 Pseudotime trajectory analysis of HCC cells

**Figure 5:**
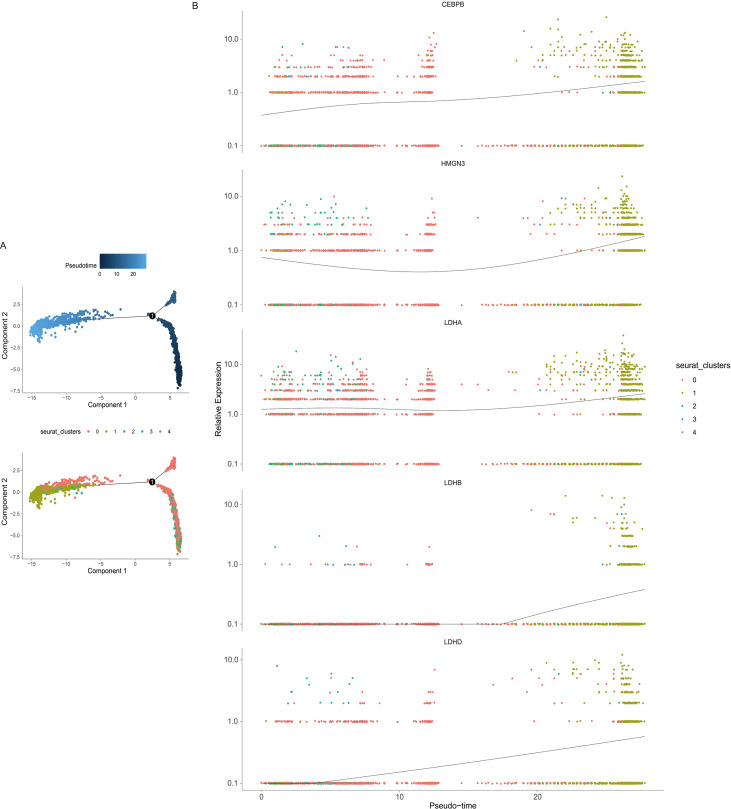
Pseudotime trajectory analysis of hepatocytes. **(A)** Pseudotime trajectory of hepatocytes from the GSE151530 dataset; darker coloration indicates a more primitive state, cells positioned right of the trajectory node represent terminally differentiated cells; post-treatment hepatocytes localize predominantly to the terminal differentiation branch. **(B)** Expression distribution of genes LDHA, LDHB, LDHD, HMGN3, and CEBPB along pseudotime; all genes exhibit an upward expression trend towards the terminal state.

Pseudotime trajectory analysis was performed on hepatocytes from the GSE151530 single-cell dataset to model their differentiation state. The inferred trajectory maps a developmental continuum, where the darker-colored cells represent the most primitive state(Left stage), and cells located at the right branch represent the terminally differentiated end-state. Our findings uncovered distinct differentiation patterns in hepatocytes before and after treatment (**Figure 5A**), with the treated cells primarily clustering in terminal differentiation branches. We subsequently examined the expression dynamics of key genes (LDHA, LDHB, LDHD, HMGN3, CEBPB) along pseudotime trajectories. The results indicated that as cells advanced toward terminal differentiation, the expression levels of these genes gradually increased (**Figure 5B**).

### 3.6 TCGA data analysis and nomogram plot

**Figure 6:**
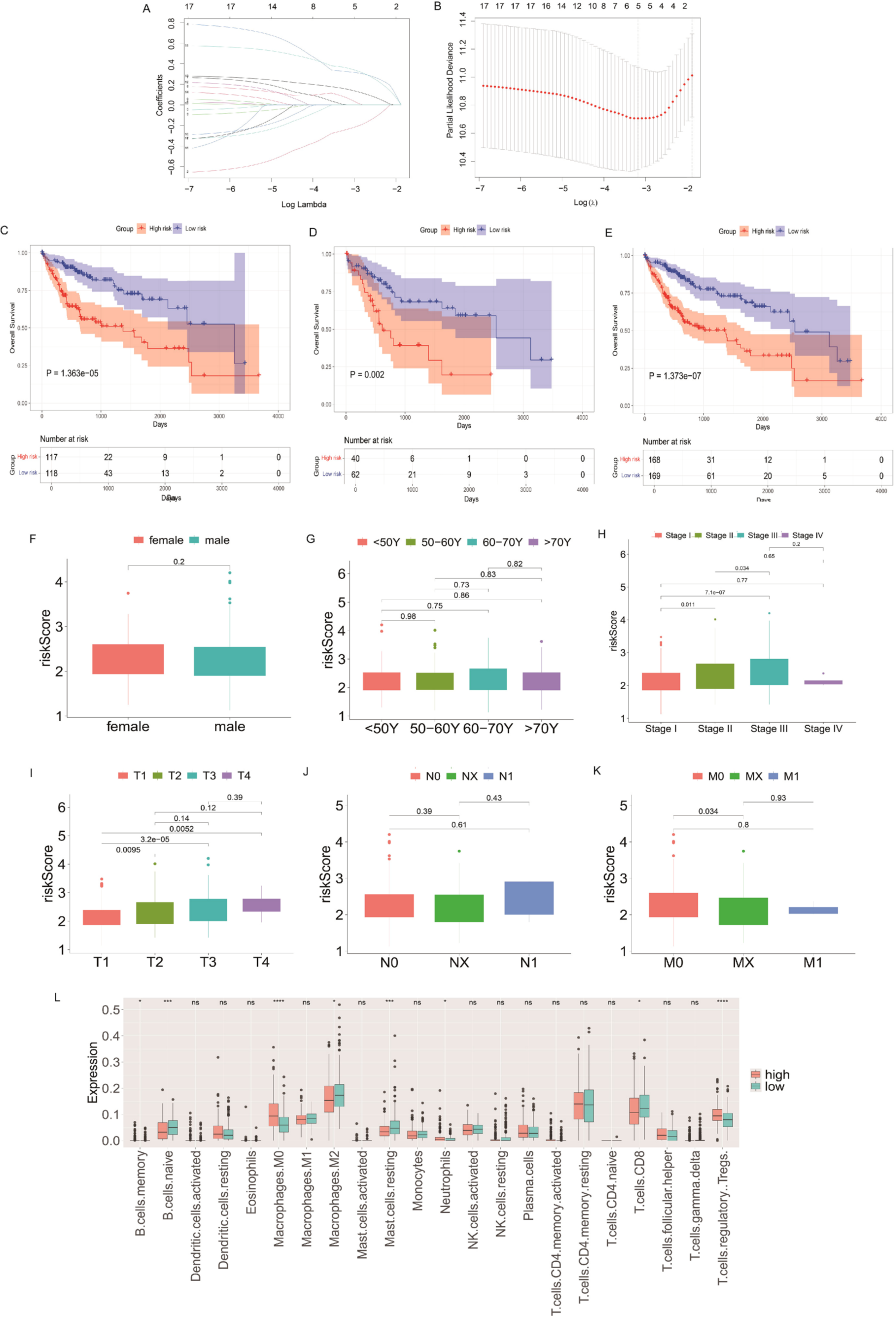
Prognostic lasso model construction, validation, and its clinical–immunological correlations in HCC. **(A, B)** Lasso coefficient profiles and cross-validation for optimal gene selection. **(C-E)** Kaplan-Meier survival analysis based on model risk score in the training cohort (70%, P = 1.363×10^-5^) **(C)**, the testing cohort (30%, P = 0.002) **(D)**, the entire TCGA-LIHC cohort (P = 1.37×10^-7^) **(E-K)** Distribution of model risk score stratified by sex **(F)**, age **(G)**, AJCC Stag **(H)**, T stage **(I)**, N stage **(J)**, and M stage **(K)**; significant differences observed for Stage and T stage, with scores increasing with disease severity. **(L)** Differences in immune cell infiltration proportions (B cells, M0 macrophages, M2 macrophages, Mast cells, Neutrophils) between high-risk and low-risk groups defined by the model score. *p < 0.05; **p < 0.01; ***p < 0.001; ****p < 0.0001.

Finally, to elucidate the critical prognostic significance of the pyruvate metabolism pathway in hepatocellular carcinoma (HCC), we employed genes within this pathway for prognostic modeling. Using Lasso regression analysis (For detailed AUC performance metrics of the model, refer to **Supplementary Figure 7**), we screened for genes significantly associated with prognosis, identifying 17 genes with a univariate Cox P-value < 0.05 (**Figures 6A, B**). A prognostic model was constructed based on these 17 genes using 70% of the data as the training cohort. Survival analysis based on the model risk score demonstrated a highly significant difference (P = 1.363e-05) in the training cohort (**Figure 6C**). This model was then validated in the remaining 30% testing cohort, where survival analysis based on the risk score also showed a significant difference (P = 0.002) (**Figure 6D**). Applying the risk score to the entire TCGA-LIHC cohort yielded an even more significant survival difference (P = 1.37×10^-7^) (**Figure 6E**).

We next investigated the association between the model risk score and key clinical parameters. No significant associations were found with age, sex, N stage, or M stage. However, the risk score showed significant associations with AJCC Stage and T stage, exhibiting a trend of increasing scores with advancing disease severity (**Figures 6F–K**). Furthermore, significant differences in the infiltration proportions of specific immune cell types—including B cells, M0 macrophages, M2 macrophages, Mast cells, and Neutrophils—were observed between the high-risk and low-risk groups defined by the model score (**Figure 6L**).

To further validate the reliability of the prognostic analysis, an external dataset (GSE14520) was employed for independent validation. A prognostic model was constructed and patients were stratified into high- and low-risk groups for survival analysis. The results demonstrated that the high-risk group exhibited significantly poorer survival outcomes compared with the low-risk group (**Supplementary Figure 8A**, P < 0.05). Subsequently, based on transcriptomic sequencing data from hepatocellular carcinoma before and after treatment, differential analysis of risk scores was performed among the untreated group, treatment-nonresponsive group, and treatment-responsive group. The results showed that the risk score in the treatment-responsive group was significantly higher than that in the untreated group (**Supplementary Figure 8B**, P < 0.05). Although the difference between the pre- and post-treatment groups yielded a P value of 0.059, the treatment-responsive group still exhibited a higher risk score than the nonresponsive group.

### 3.7 Independent prognostic value of pyruvate metabolism signature in HCC validated

**Figure 7:**
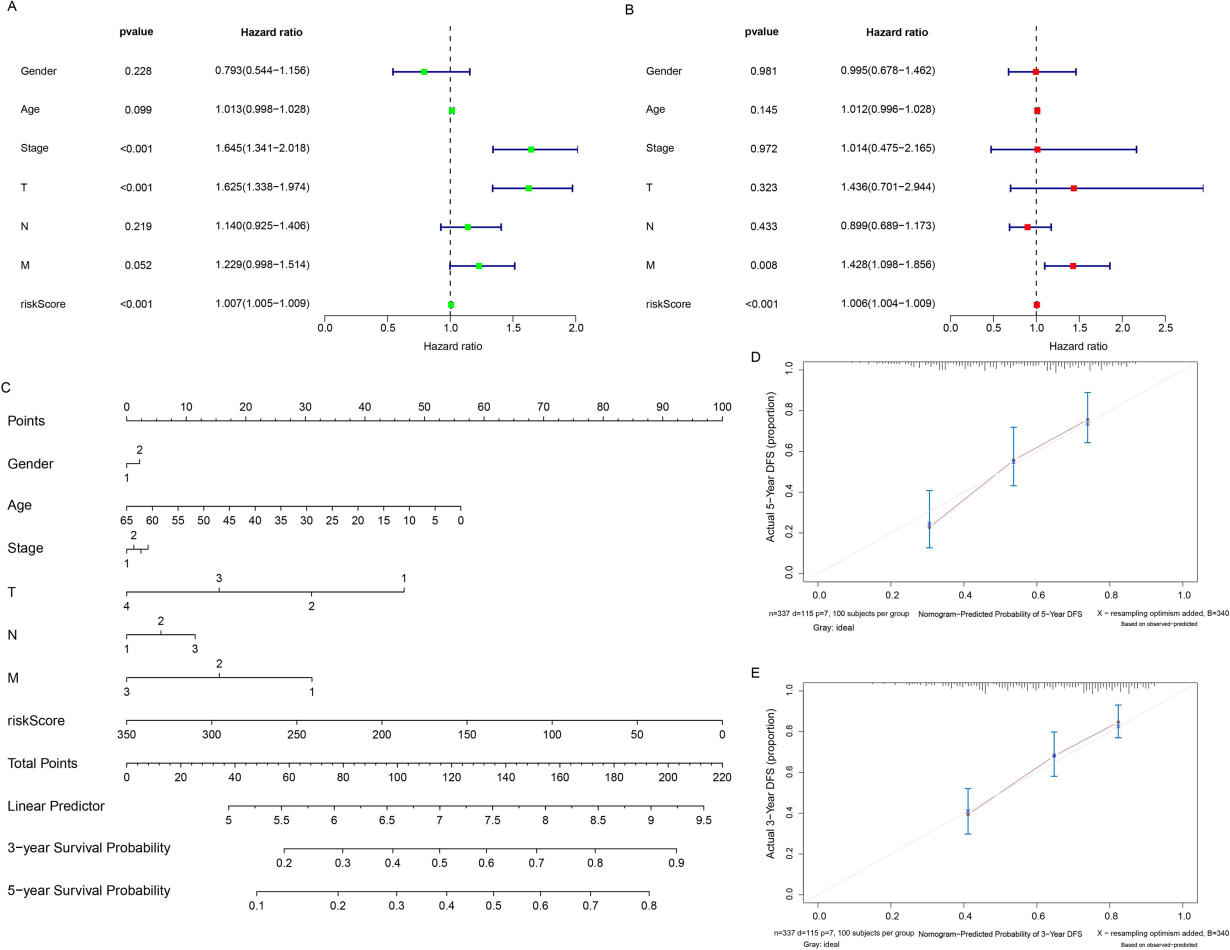
Univariate/multivariate prognostic analysis and nomogram construction. **(A)** Forest plot of univariate Cox regression analysis for prognostic factors. **(B)** Forest plot of multivariate Cox regression analysis for independent prognostic factors. **(C)** Prognostic nomogram incorporating the model risk score and clinical factors. **(D, E)** Calibration curves for the nomogram-predicted versus actual 3-year and 5-year overall survival probabilities.

Univariate and multivariate Cox regression analyses confirmed that the model risk score served as a significant independent prognostic factor (**Figures 7A, B**). To facilitate clinical application, a nomogram was constructed incorporating the risk score and relevant clinical factors. The nomogram demonstrated that the risk score was the primary contributor to prognostic evaluation (**Figure 7C**). Calibration curves further indicated good concordance between the nomogram-predicted survival probabilities and actual observed outcomes, confirming its reliability (**Figures 7D, E**). Collectively, these analyses robustly establish our pyruvate metabolism-based signature as an independent risk factor for HCC patient prognosis.

### 3.8 The combination therapy demonstrates the best anti-tumor effect with a good safety profile

**Figure 8:**
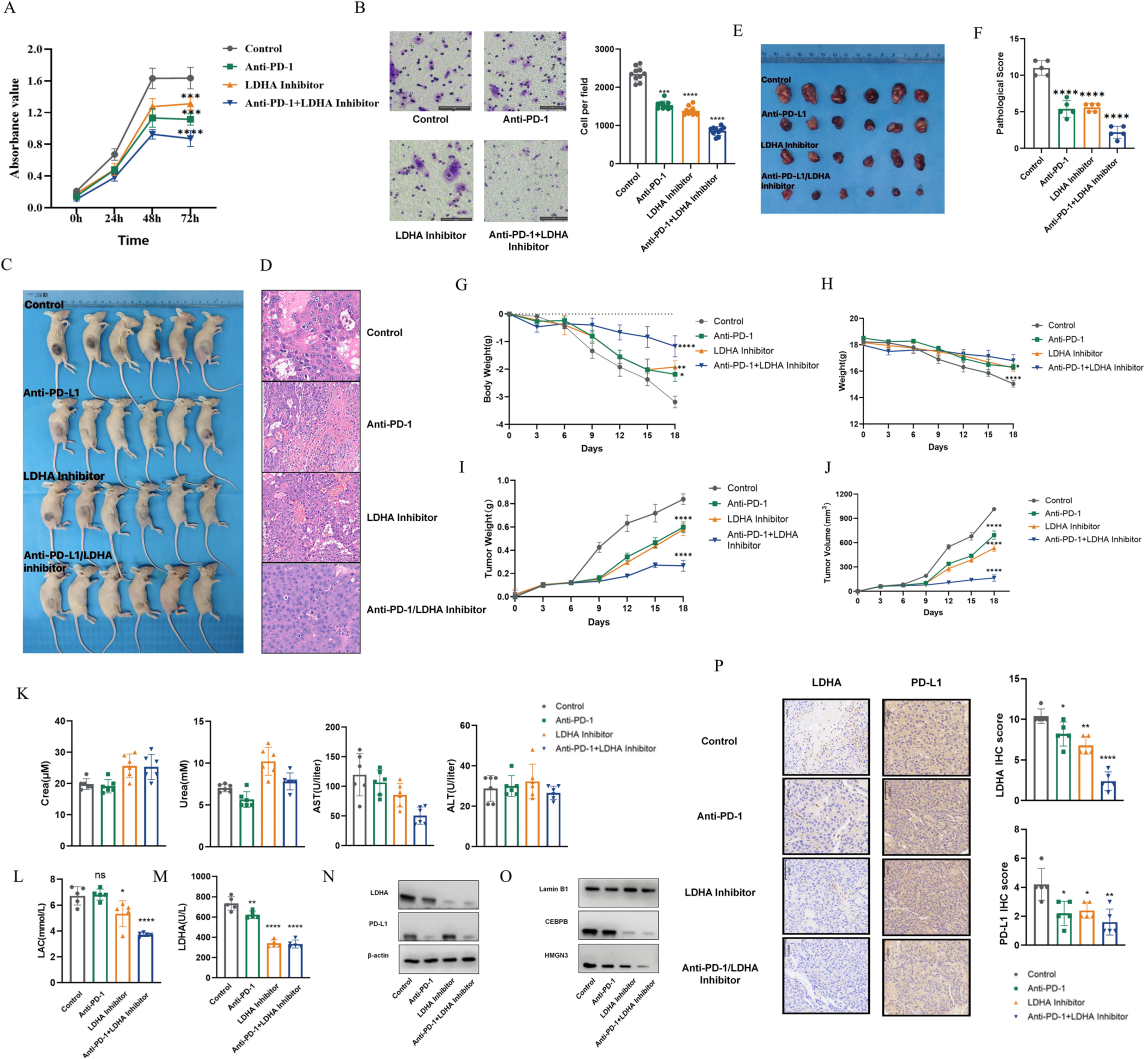
Combination therapy shows a significant anti-tumor effect and a good safety profile. **(A)** CCK8 assay of Huh7 cells. **(B)** Transwell migration assay of Huh7 cells. **(C, D)** Subcutaneous xenograft tumors in mice and HE staining of tumors. **(E)** Comparison of subcutaneous tumor volumes. **(F)** Pathological scoring of subcutaneous tumors. **(G)** Changes in mouse body weight. **(H)** Relative changes in mouse body weight. **(I)** Changes in subcutaneous tumor weight. **(J)** Changes in subcutaneous tumor volume. **(K)** Comparison of biochemical indicators in mouse orbital blood. **(L, M)** Differential expression of LAC **(L)** and LDHA **(M)** among the treatment groups. **(N)** Western blot analysis of PD-L1 and LDHA protein expression in different treatment groups. **(O)** Western blot analysis of nuclear expression levels of HMGN3 and CEBPB in different treatment groups (Lamin B1 used as the nuclear loading control). **(P)** IHC staining of tumor tissues showing LDHA and PD-L1 expression across different treatment groups. Scale bar: 40 μm. *p < 0.05; **p < 0.01; ***p < 0.001; ****p < 0.0001.

First, we cultured Huh7 cells (**Figure 8A**) and HepG2 cells (**Supplementary Figure 9A**) *in vitro* and used the CCK8 assay to detect cell growth under treatment with PD-1 antibody alone, the LDHA small molecule inhibitor ((R)-GNE-140) alone, or the combination of PD-1 antibody and (R)-GNE-140. As shown in **Figure 8A**, cells in all four groups reached a plateau at 48h. Furthermore, the combination therapy group exhibited the poorest cell growth, and this difference was statistically significant. *In vitro* Transwell chamber assays revealed that both PD-1 antibody and (R)-GNE-140 could effectively inhibit the migration of Huh7 cells (**Figure 8B**, P<0.05) and HepG2 cells (**Supplementary Figure 9B**, P<0.05), which, to a certain extent, suppressed the malignant phenotype of HCC.

Next, we conducted a subcutaneous xenograft tumor experiment in nude mice (**Figure 8C**). When the tumors grew to approximately 100 mm³, intraperitoneal (i.p.) administration was initiated, three times a week for three weeks. On the 18th day after the initial administration, we collected the subcutaneous tumors and mouse orbital blood, and the subcutaneous tumors were subjected to HE staining (**Figures 8D, E**). Pathological scoring showed that the combination therapy group had the lowest pathological score, indicating the greatest extent of tumor necrosis in this group (**Figure 8F**).

Throughout the administration process, no significant changes in body weight were observed in the three treatment groups (**Figure 8I**). Only the untreated control group showed a significant decrease in body weight, likely due to tumor cachexia. In our calculation of the relative change rate of mouse body weight, the weight loss in the control group was more pronounced (**Figure 8J**). In contrast, the tumor volume and weight in the combination therapy group were significantly smaller than those in the monotherapy groups and the control group, demonstrating a significant anti-tumor advantage for the combination therapy (**Figures 8G, H**). In summary, the combination therapy group exhibited a significant anti-tumor effect without causing substantial impacts on the liver and kidney functions of the mice. Biochemical analysis of the mouse orbital blood revealed that liver function (ALT/AST) and kidney function (BUN/Cre) indicators for all groups were within the normal range. No significant elevation was observed in the combination group (**Figure 8K**).

Furthermore, the expression levels of LAC and LDHA were evaluated across different treatment groups in Huh7 cells and HepG2 cells. The results showed that both lactate levels and LDHA enzymatic activity were significantly reduced in the combination therapy group (anti-PD-1 + LDHA inhibitor) (**Figures 8L, M**; **Supplementary Figures 9C, D**; P < 0.05). Western blot analysis demonstrated that both LDHA inhibitor monotherapy and the combination treatment markedly downregulated LDHA and PD-L1 protein expression, with a more pronounced reduction observed in the combination group (**Figure 8N**; **Supplementary Figure 9E**). In addition, the nuclear expression of the upstream regulators HMGN3 and CEBPB, which modulate LDHA activity, was further examined. The results revealed that combination therapy significantly decreased the nuclear localization of HMGN3 and CEBPB (**Figure 8O**, **Supplementary Figure 9F**), consistent with the findings in the HepG2 cell line, suggesting that the HMGN3/CEBPB–LDHA regulatory axis may represent a conserved mechanism. Moreover, immunohistochemical (IHC) staining of xenograft tumors showed that LDHA and PD-L1 positive scores were significantly lower in the combination therapy group compared with the monotherapy and control groups, with a more pronounced reduction of PD-L1 expression observed in the Huh7 model (**Figure 8P**; **Supplementary Figure 9G**; P < 0.05). Collectively, these findings further support the synergistic inhibitory effect of the combination therapy *in vivo*.

At the end of the experiment, the mice were euthanized via an intraperitoneal injection of pentobarbital at a dose of 150 mg/kg, which first induced deep anesthesia followed by respiratory arrest.

## 4 Discussion

In this study, we employed single-cell analysis for the first time to systematically investigate the dynamic alterations in energy metabolism of HCC patients before and after immune checkpoint inhibitor (ICI) treatment and its potential impact on drug resistance. Our findings revealed that post-ICI therapy, genes related to pyruvate metabolism, such as LDHA, LDHB, and LDHD, were significantly upregulated. This was accompanied by decreased glycolytic activity and suppressed lactate efflux pathways, leading to intracellular lactate accumulation within tumor cells and the formation of an acidic microenvironment, thereby inhibiting T-cell function. Concurrently, intracellular TCA cycle activity and protein acetylation levels markedly decreased, while the AMPK signaling pathway was significantly activated, suggesting that tumor cells prioritize ATP generation to sustain survival and enhance resistance capabilities under energy-restricted conditions. At the level of post-translational modifications, we observed dynamic changes in the expression of lactylation and acetylation “writers” and “erasers.” Additionally, DNA repair-related genes MRE11A and NBN were significantly upregulated in resistant subpopulations, implying that post-translational modifications may enhance DNA repair by promoting heterochromatin relaxation, thereby aiding tumor cells in resisting drug-induced damage. Regarding transcriptional regulation, transcription factors (TFs) such as MYC, SREBF1, and CEBPB exhibited differential high expression. Validation using Cistrome DB confirmed their direct regulation of various metabolic genes, providing refined transcriptional-level evidence for the resistance mechanism. Furthermore, cell-cell communication network analysis demonstrated significantly enhanced interactions along the SPP1-CD44 and APOA1-ABCA1 ligand-receptor axes between hepatocytes and T cells/tumor-associated macrophages (TAMs), suggesting that metabolic signaling plays a pivotal role within the immune microenvironment and may represent novel therapeutic targets. Pseudotime trajectory analysis indicated that resistant cells exhibit terminal differentiation features, with pyruvate metabolism gene expression progressively increasing along the differentiation path. A prognostic model constructed based on these genes independently predicted patient survival in the TCGA-LIHC cohort and correlated significantly with clinical stage and immune infiltration. Furthermore, our *in vitro* and *in vivo* experiments provided direct validation for these findings, demonstrating that combination therapy with a PD-1 antibody and the specific LDHA inhibitor ((R)-GNE-140) significantly inhibited tumor (Huh7) proliferation and migration, suppressed xenograft tumor growth, and exhibited a good safety profile.

The tumor microenvironment (TME) constitutes a complex ecosystem where interactions between tumor cells, immune cells, and stromal cells profoundly influence tumor response to immune checkpoint inhibitors (ICIs). Pyruvate metabolism, a critical node in cellular energy metabolism, plays vital roles in both tumor and immune cells. Accumulating evidence indicates that aberrant remodeling of pyruvate metabolism is a key mechanism underlying ICI resistance in tumors (25). Specifically, to meet the demands of rapid proliferation, tumor cells often favor aerobic glycolysis (the Warburg effect), converting glucose to pyruvate and subsequently to lactate even under oxygen-sufficient conditions, rather than entering the tricarboxylic acid (TCA) cycle. This metabolic reprogramming not only provides rapidly proliferating tumor cells with biosynthetic precursors but also acidifies the TME, thereby inhibiting the anti-tumor activity of T cells and promoting the infiltration of immunosuppressive cells (26), ultimately leading to suboptimal ICI efficacy or resistance. Moreover, pyruvate metabolites like lactate can directly impact immune cell function, for instance, by inhibiting dendritic cell maturation and antigen-presenting capacity (27), further exacerbating immunosuppression. In hepatocellular carcinoma (HCC), the dysregulation of pyruvate metabolism is particularly prominent in ICI resistance, with members of the lactate dehydrogenase (LDH) family, especially LDHA, LDHB, and LDHD, playing crucial roles. LDHA primarily catalyzes the reduction of pyruvate to lactate, acting as the terminal enzyme of glycolysis. Its overexpression is closely associated with malignant progression and immune evasion in various cancers (28). In HCC, LDHA overexpression promotes lactate production, leading to TME acidification which subsequently impairs CD8+ T-cell function, hindering their ability to effectively kill tumor cells and thus contributing to ICI resistance (29). Conversely, LDHB primarily catalyzes the oxidation of lactate to pyruvate and is generally considered a tumor suppressor; its reduced expression may contribute to lactate accumulation (30). LDHD, a D-lactate dehydrogenase distinct from L-lactate dehydrogenases (LDHA and LDHB), has a more complex role but has also been implicated in metabolic reprogramming and immunosuppression in certain cancers (31). Collectively, high LDHA expression and its mediated lactate accumulation are key factors driving ICI resistance in HCC, while imbalances in LDHB and LDHD may also promote HCC resistance to ICIs by disrupting the pyruvate-lactate equilibrium. This aligns with our single-cell data showing the involvement of LDHA, LDHB, and LDHD in pyruvate metabolism-mediated ICI resistance. Our subsequent *in vitro* and *in vivo* combination therapy experiments, which used the LDHA inhibitor (R)-GNE-140, provided strong supporting evidence, confirming that targeting LDHA can effectively inhibit tumor growth and migration, thereby enhancing the anti-tumor effect of PD-1 blockade.

Our experimental data revealed decreased protein acetylation levels alongside reduced TCA cycle scores, indicating insufficient cellular energy supply. This energy-deficient state was further corroborated by the activation of the AMPK signaling pathway. AMPK (AMP-activated protein kinase), a key regulator of cellular energy homeostasis, is activated when the ATP/AMP ratio decreases, promoting catabolic pathways for energy production and inhibiting anabolic pathways to conserve energy (32). Under drug pressure, cells may prioritize channeling acetyl-CoA into the TCA cycle to meet urgent energy demands, consequently reducing the pool of acetyl-CoA available for protein acetylation. This is consistent with our observed decrease in protein acetylation levels. Such metabolic flux adjustments in response to cellular stress have been reported in various contexts (25, 33).

Notably, our study also uncovered complex crosstalk between lactylation and acetylation. Dynamic changes were observed in the expression of lactylation and acetylation “writers” and “erasers” before and after treatment, with some “writer” genes upregulated post-treatment. This suggests cells may fine-tune protein modification states by enhancing the activity of specific enzymes. More importantly, we observed high expression of DNA repair genes like MRE11A and NBN. MRE11A and NBN are key components of the DNA double-strand break repair pathway, and their upregulation typically signifies active DNA damage repair (34). We speculate that acetylation and lactylation may enhance DNA repair efficiency by directly modifying these DNA repair-associated proteins, such as histones or non-histone proteins, thereby aiding hepatocytes in resisting drug-induced damage stress. For instance, histone acetylation can alter chromatin structure, making damaged DNA sites more accessible to repair enzymes (35). Lactylation, an emerging post-translational modification, has also been implicated in the DNA damage response (12). Therefore, acetylation and lactylation may act synergistically to shape cellular tolerance to drug pressure.

Our analysis further identified a set of key transcription factors differentially expressed in hepatocytes post-treatment, including MYC, SP5, HLF, SREBF1, LEF1, HMGN3, and CEBPB. These TFs are typically involved in regulating cell proliferation, differentiation, metabolism, and stress responses (36). For example, MYC is a well-known oncogene regulating cell cycle, metabolism, and apoptosis (37). SREBF1 is a master regulator of fatty acid and cholesterol synthesis (38). CEBPB plays a role in liver metabolism and inflammatory responses (39). Their differential expression indicates a remodeling of the transcriptional regulatory network in response to drug-induced metabolic stress. Combined with validation from Cistrome DB, we found direct regulatory relationships between these differentially expressed TFs and pyruvate metabolism-related genes, such as LDHA and AARS. LDHA (Lactate Dehydrogenase A) is the key enzyme catalyzing the conversion of pyruvate to lactate at the terminus of glycolysis. Its activity is frequently upregulated in various cancers to support the energy demands of rapidly proliferating cells (Warburg effect) (40). AARS (Alanyl-tRNA Synthetase) primarily participates in protein synthesis but has also been linked to metabolic reprogramming in certain contexts (41). Our experiments demonstrated that the combination of anti-PD-L1 inhibitors and LDHA inhibitors significantly suppressed lactate levels, with HMGN3 and CEBPB expression levels exhibiting the same therapeutic response as LDHA.

The regulation of genes like LDHA and AARS by TFs (HMGN3 and CEBPB) further supports our conclusion that HCC cells adapt to drug pressure by precisely modulating the pyruvate metabolism pathway. For instance, by upregulating LDHA expression, cells can increase lactate production, sustaining a high glycolytic flux even under normoxic conditions. This provides rapid ATP supply and generates intermediates for anabolic processes, helping cells cope with drug-induced energy depletion and biosynthetic demands. Such TF-mediated metabolic adaptability may constitute a crucial mechanism underlying the development of treatment resistance in HCC cells.

Our study delved into the dynamic changes within the tumor microenvironment (TME) before and after drug treatment, particularly the remodeling of cell-cell communication networks. Results showed a significant increase in communication weight between hepatocytes post-treatment, possibly reflecting enhanced intercellular interactions under stress to coordinate coping mechanisms or adaptive responses. More specifically, we observed strengthened specific ligand-receptor interactions between hepatocytes and immune cells, particularly T cells and tumor-associated macrophages (TAMs). Interactions along the SPP1-CD44 and APOA1-ABCA1 axes were notably prominent.

SPP1 (Osteopontin) is a multifunctional cytokine playing key roles in tumor progression, metastasis, and immune regulation (42). CD44, one of its primary receptors, is expressed on various tumor and immune cells (43). Activation of the SPP1-CD44 axis has been shown to promote tumor cell proliferation, migration, and invasion, while also influencing immune cell function—such as suppressing T-cell activity and promoting M2 polarization of TAMs—thereby fostering an immunosuppressive microenvironment (44). Our findings suggest that post-treatment, hepatocytes may mediate immune evasion and resistance by upregulating SPP1 expression or enhancing its binding to CD44 on T cells and TAMs.

On the other hand, APOA1 (Apolipoprotein A1) is primarily involved in lipid metabolism, and ABCA1 (ATP-Binding Cassette Transporter A1) is a key transporter for cholesterol efflux (45). Although the role of the APOA1-ABCA1 axis in the TME is less studied, evidence suggests lipid metabolism significantly impacts immune cell function and tumor progression (46). Enhanced APOA1-ABCA1 interaction between hepatocytes and TAMs may indicate that under drug pressure, cells modulate lipid metabolism to influence immune cell phenotype and function, thereby affecting therapeutic efficacy.

Furthermore, our analysis revealed that a series of important metabolic genes and transcription factors, including LDHA, LDHB, LDHD, HMGN3, and CEBPB, exhibited a trend towards high expression along the cellular differentiation trajectory. LDHA, LDHB, and LDHD are members of the lactate dehydrogenase family, catalyzing the reversible conversion between pyruvate and lactate (47). LDHA is typically highly expressed in cancer cells to support glycolysis, while LDHB and LDHD play diverse roles depending on cell type and metabolic context (48). Their high expression at the terminal differentiation stage further underscores the centrality of pyruvate metabolism in HCC cell adaptation to drug pressure. HMGN3 (High Mobility Group Nucleosomal Binding Domain 3) participates in chromatin remodeling and gene expression regulation (49), and CEBPB (CCAAT/Enhancer-Binding Protein Beta) plays critical roles in liver development, metabolism, and stress responses (39). The temporally coordinated high expression of these genes and TFs along the differentiation trajectory strongly suggests their involvement in the precise temporal regulation of metabolic reprogramming and resistance acquisition in HCC cells.

Finally, building upon our in-depth investigation of pyruvate metabolism-related genes, we successfully constructed a prognostic signature and validated it in an independent TCGA (The Cancer Genome Atlas) HCC cohort. Results demonstrated that this prognostic signature possesses significant prognostic value, effectively stratifying patients into different risk groups. This indicates that alterations in pyruvate metabolism are not only a key mechanism for HCC cells coping with drug pressure but also a potential biomarker for predicting patient survival. Importantly, the signature risk score correlated with the infiltration proportions of various immune cells, including B cells, M0 macrophages, M2 macrophages, mast cells, and neutrophils.

In summary, this study, through integrated multi-omics analysis, particularly the application of single-cell RNA sequencing combined with bulk transcriptomic data, provides unprecedented cellular resolution into the heterogeneity and metabolic reprogramming of HCC before and after ICI therapy. Traditional bulk sequencing struggles to capture fine-grained changes within specific TME cell subpopulations. The introduction of single-cell sequencing enabled us to resolve the transcriptomic features of specific cell types (e.g., hepatocytes), revealing therapy-induced cell fate transitions and metabolic adaptations. Mechanistically, leveraging cell communication analysis, we uncovered novel ligand-receptor interactions (e.g., SPP1-CD44, APOA1-ABCA1) between hepatocytes and T cells/TAMs post-treatment, interactions that may play crucial roles in immunosuppression and resistance mechanisms. Pseudotime trajectory analysis, for the first time, revealed the transition of hepatocytes towards a terminal differentiation state post-treatment, offering a new perspective for understanding cellular adaptation under drug pressure. Furthermore, TF analysis precisely delineated the roles of key regulators like MYC, SREBF1, and CEBPB in controlling pyruvate metabolism-related genes (LDHA, LDHB, LDHD), elucidating how HCC cells maintain metabolic homeostasis through a sophisticated transcriptional regulatory network in response to drug stress. These in-depth mechanistic explorations extend beyond simple correlative analyses, painting a clearer picture of the molecular underpinnings of ICI resistance. Finally, we constructed and validated a pyruvate metabolism-based prognostic signature in the independent TCGA cohort. This signature not only accurately predicts HCC patient prognosis but also correlates with clinical features and immune infiltration, highlighting its substantial potential as an independent prognostic factor.

Despite these significant advances, this study has limitations. Firstly, the sample size of the single-cell datasets is relatively limited, particularly the number of paired samples (less than 10 pairs). This may constrain statistical power for detecting differences in rare cell subpopulations or subtle effects. Although we validated key findings in larger bulk sequencing cohorts, future studies require increased single-cell paired sample sizes to further bolster the reliability of conclusions. Secondly, while this study successfully validated the therapeutic potential of targeting LDHA *in vitro* and *in vivo* using the inhibitor (R)-GNE-140, several proposed mechanisms, such as the crosstalk between protein acetylation/lactylation and DNA repair, and the regulatory roles of specific TFs on pyruvate metabolism, require further *in vitro* and *in vivo* experimental validation. For instance, employing gene knockout/overexpression techniques to alter LDHA, HMGN3, or CEBPB expression and observing the impact on HCC cell metabolic behavior and resistance under drug pressure; or directly intervening with metabolites like lactate or acetyl-CoA to validate their role in resistance. Such experiments would provide more direct causal evidence strengthening our conclusions. Additionally, clinical validation of the constructed prognostic signature necessitates large-scale, multi-center prospective clinical cohorts. This will not only further confirm its validity but also assess its generalizability across diverse populations and treatment regimens. Based on our findings, which demonstrate that targeting pyruvate metabolism (specifically LDHA) with (R)-GNE-140 in combination with PD-1 inhibition is a promising strategy, future research holds promise for developing novel therapeutic strategies targeting pyruvate metabolism or lactate clearance to overcome ICI resistance in HCC. For example, investigating LDHA inhibitors or lactate transporter (MCTs) inhibitors could offer new treatment options for ICI-resistant patients. Finally, the immunological background of the nude mouse model used in this study has certain limitations. For instance, nude mice lack a complete T-cell immune background and do not fully match the clinical immune background of immune checkpoint inhibitors, which still requires further research and supplementation in our future studies.

Future research should further investigate the differential effects of various immune checkpoint inhibitor (ICI) regimens on energy metabolism in hepatocellular carcinoma (HCC), such as combinations of PD-L1/CTLA-4 inhibitors with targeted agents compared with single-agent PD-1 blockade. Clarifying these differences may help optimize therapeutic strategies for patients. Our findings provide preliminary support for this concept. Specifically, (R)-GNE-140, an LDHA inhibitor, reduces tumor lactate levels and alleviates immunosuppression. When combined with PD-1 blockade, it synergistically modulates lactate metabolism, highlighting its potential for further clinical translation. In addition, future studies should perform higher-resolution analyses of non-hepatocyte populations within the tumor microenvironment (TME), including B cells, myeloid cells, and endothelial cells, to characterize their metabolic reprogramming and communication with hepatocytes. Such investigations may provide a more comprehensive understanding of the mechanisms underlying ICI resistance in HCC. In conclusion, our work offers novel insights into the mechanisms of ICI resistance in HCC and identifies potential therapeutic targets for future strategy development, although it requires refinement through larger-scale studies and deeper mechanistic validation.

## Data Availability

The original contributions presented in the study are included in the article/**Supplementary Material**. Further inquiries can be directed to the corresponding authors.

## Supplementary material

Supplementary Figure 1 The decontX histogram of contamination fraction removal for environmental RNA (0.3 threshold).

Supplementary Figure 2 Comparison of PCA analysis with and without inclusion of cell cycle scores.

Supplementary Figure 3 The differential expression of CD274 and CTLA4 between treated and untreated cell types.

Supplementary Figure 4 Comparison of protein acetylation and tricarboxylic acid (TCA) cycle (TCA) score differences among different hepatocyte subpopulations (clusters 0-4), with protein acetylation scores on the left and TCA scores on the right.

Supplementary Figure 5 GSVA-based analysis of protein acetylation and TCA cycle activity in single-cell atlases.

Supplementary Figure 6 Analysis of differences in pathway scores across different cell subpopulations before and after treatment.

Supplementary Figure 7 The ROC curve is used to evaluate the reliability of the Lasso model, where an area under the curve (AUC) closer to 1.0 indicates better discriminative performance of the model for prognostic outcomes.

Supplementary Figure 8 External dataset validation. **(A)** The external dataset GSE14520 from the HCCDB database was incorporated to construct a prognostic model, and survival analysis was performed based on stratification into high- and low-risk score groups. **(B)** Bulk RNA-seq data of hepatocellular carcinoma before and after treatment were obtained, and differential analysis of risk scores was conducted among the untreated, treatment-nonresponsive, and treatment-responsive groups. A P value < 0.05 was considered statistically significant.

Supplementary Figure 9 *In vitro* and *in vivo* functional validation in HepG2 cells. **(A)** Cell viability of HepG2 cells in different treatment groups measured by CCK-8 assay. **(B)** Transwell migration assay evaluating the migratory capacity of HepG2 cells across different treatment groups. **(C, D)** Differential expression of LAC **(C)** and LDHA **(D)** among the treatment groups. **(E)** Western blot analysis of PD-L1 and LDHA protein expression in different treatment groups. **(F)** Western blot analysis of nuclear expression levels of HMGN3 and CEBPB in different treatment groups (Lamin B1 used as the nuclear loading control). **(G)** IHC staining of tumor tissues showing LDHA and PD-L1 expression across different treatment groups. Scale bar: 100μm. *p < 0.05; **p < 0.01; ***p < 0.001; ****p < 0.0001.
