# Supplementary figures and images for: The inhibitory effect of hepatic cancer energy metabolism on immune checkpoint therapy: perspectives from single-cell multi-omics analysis
Source: Front Immunol. 2026 Apr 13;17:1753670. doi: 10.3389/fimmu.2026.1753670 (PMC13111318; doi:10.3389/fimmu.2026.1753670)

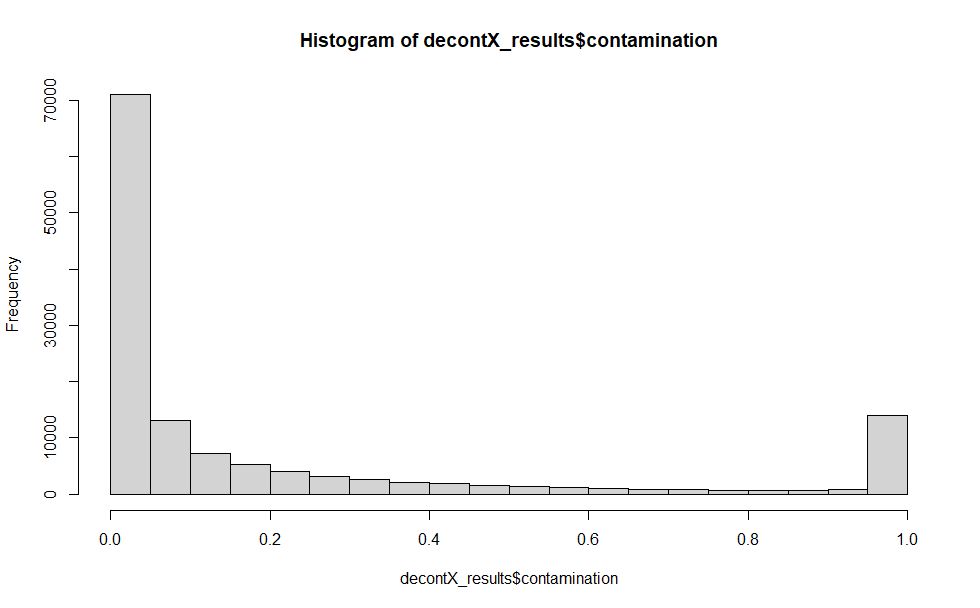

Supplement: Supplementary Figure 1 — The decontX histogram of contamination fraction removal for environmental RNA (0.3 threshold). [file Image1.jpeg]

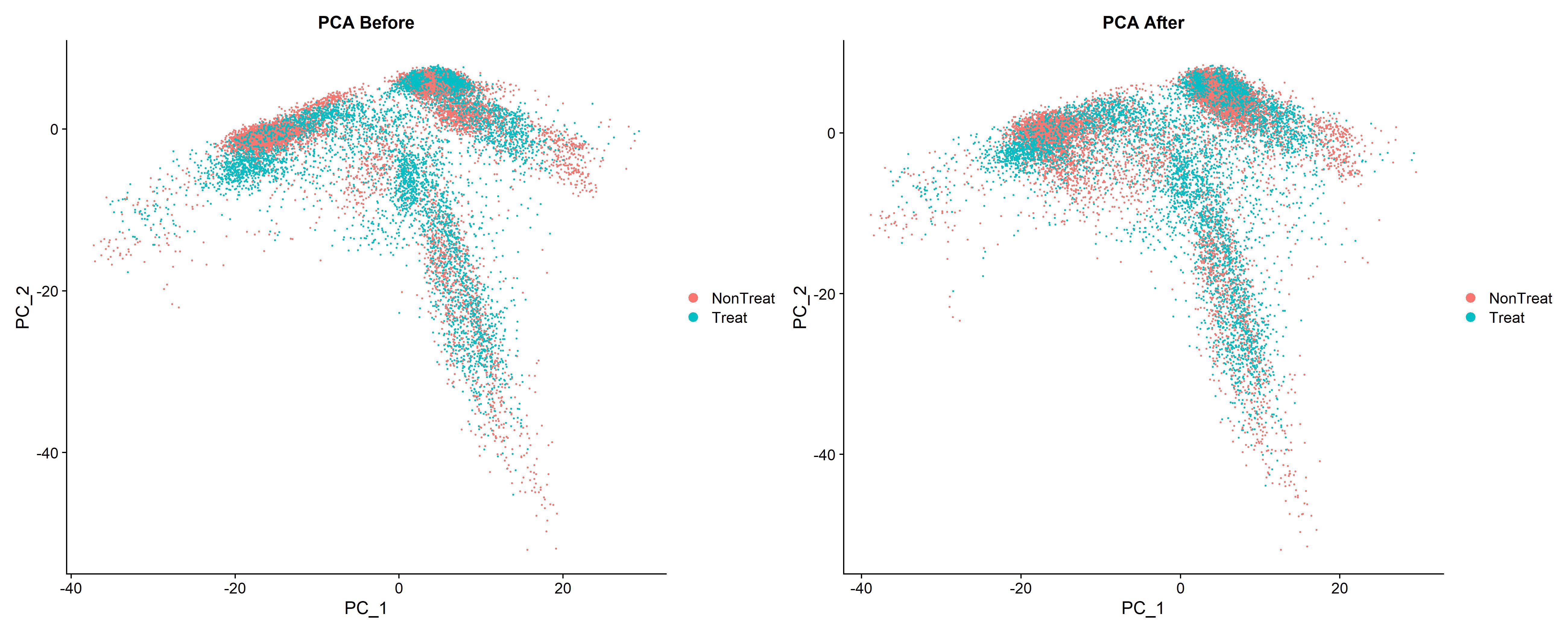

Supplement: Supplementary Figure 2 — Comparison of PCA analysis with and without inclusion of cell cycle scores. [file Image2.jpeg]

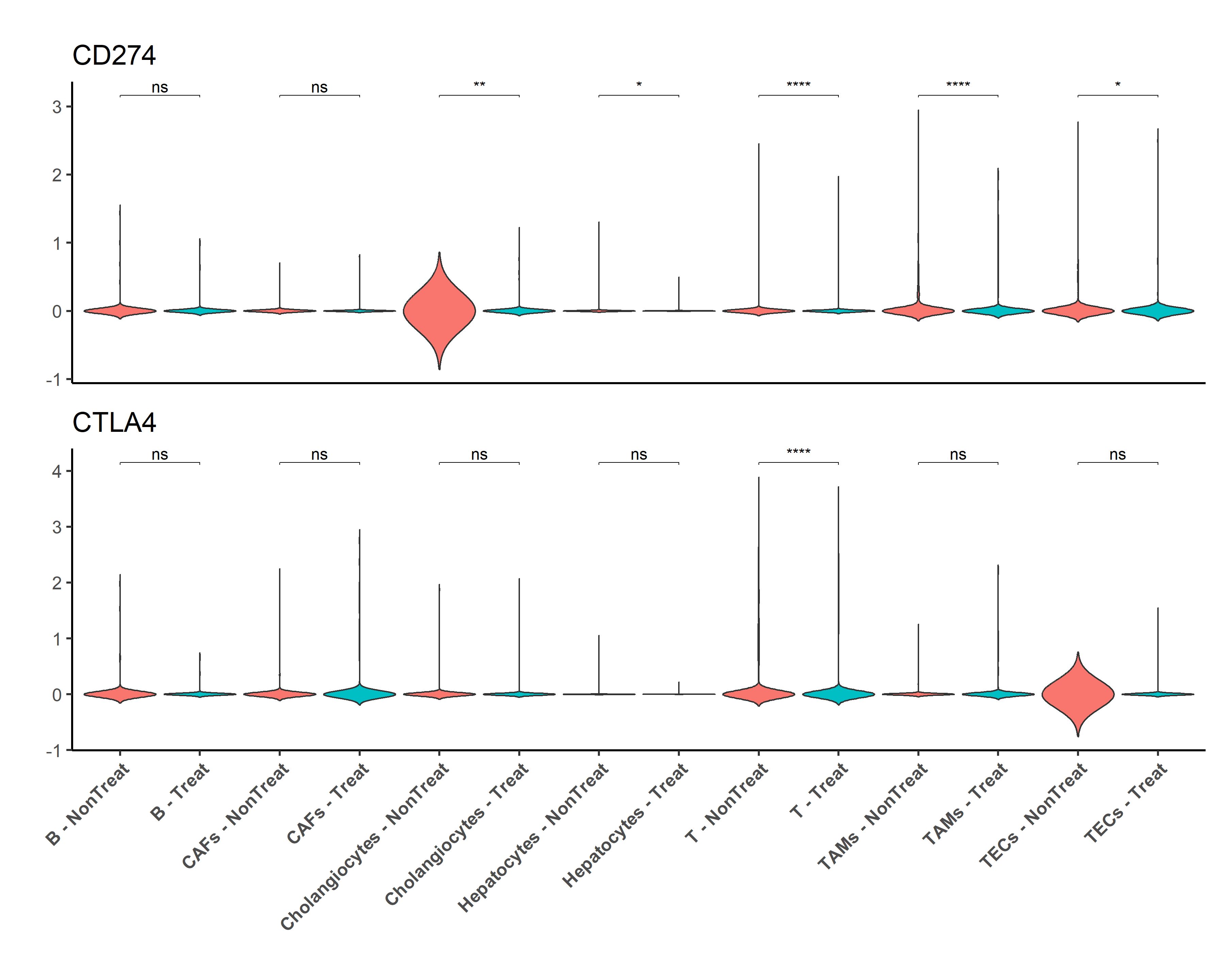

Supplement: Supplementary Figure 3 — The differential expression of CD274 and CTLA4 between treated and untreated cell types. [file Image3.jpeg]

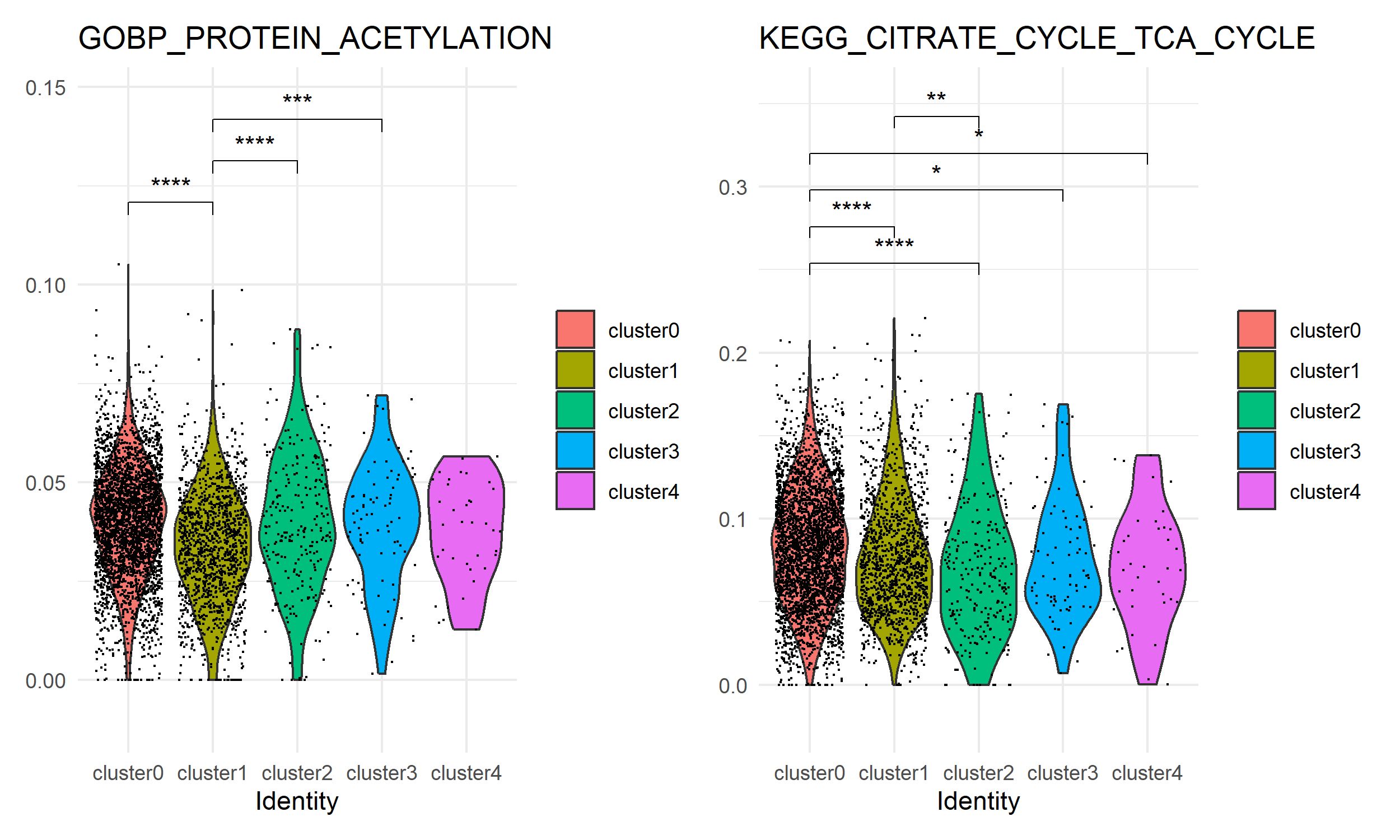

Supplement: Supplementary Figure 4 — Comparison of protein acetylation and tricarboxylic acid (TCA) cycle (TCA) score differences among different hepatocyte subpopulations (clusters 0-4), with protein acetylation scores on the left and TCA scores on the right. [file Image4.jpeg]

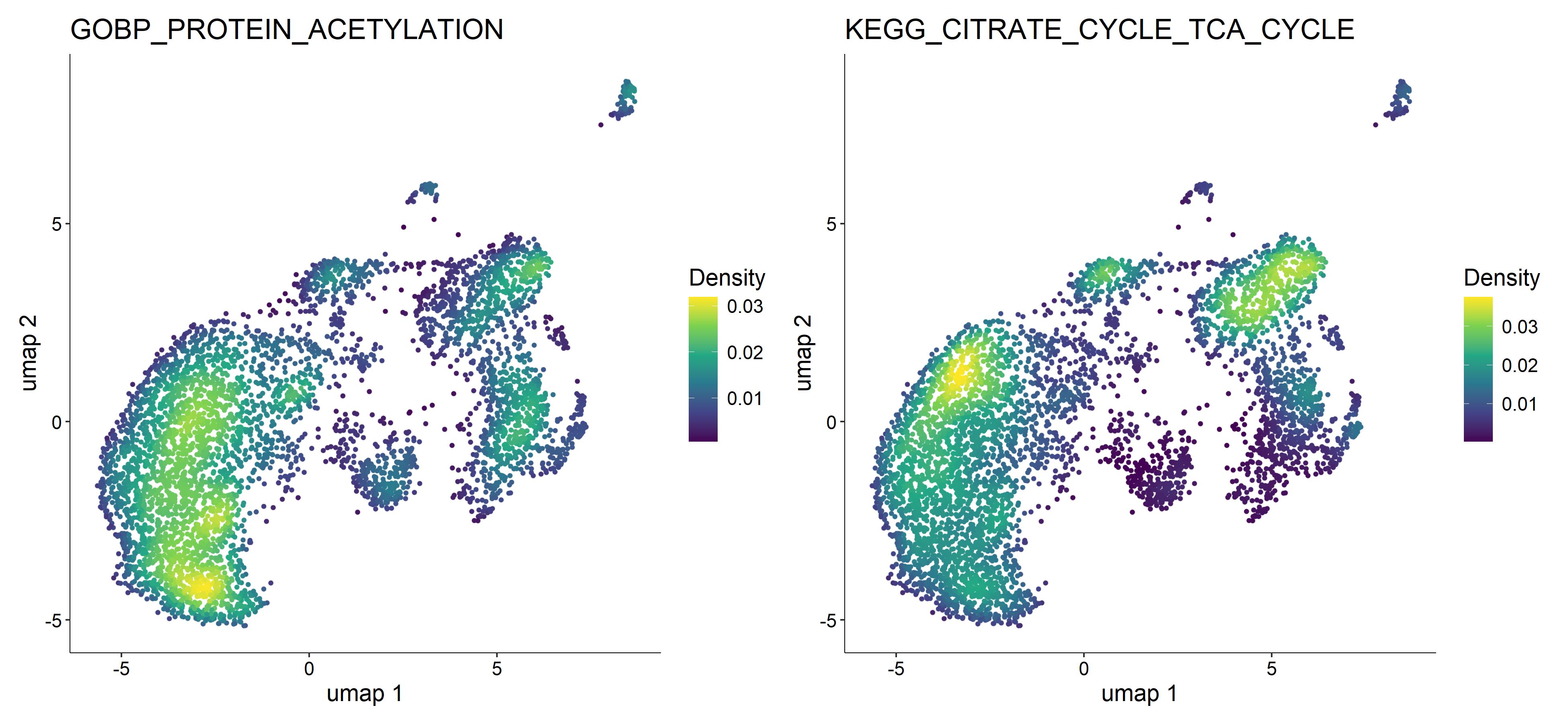

Supplement: Supplementary Figure 5 — GSVA-based analysis of protein acetylation and TCA cycle activity in single-cell atlases. [file Image5.jpeg]

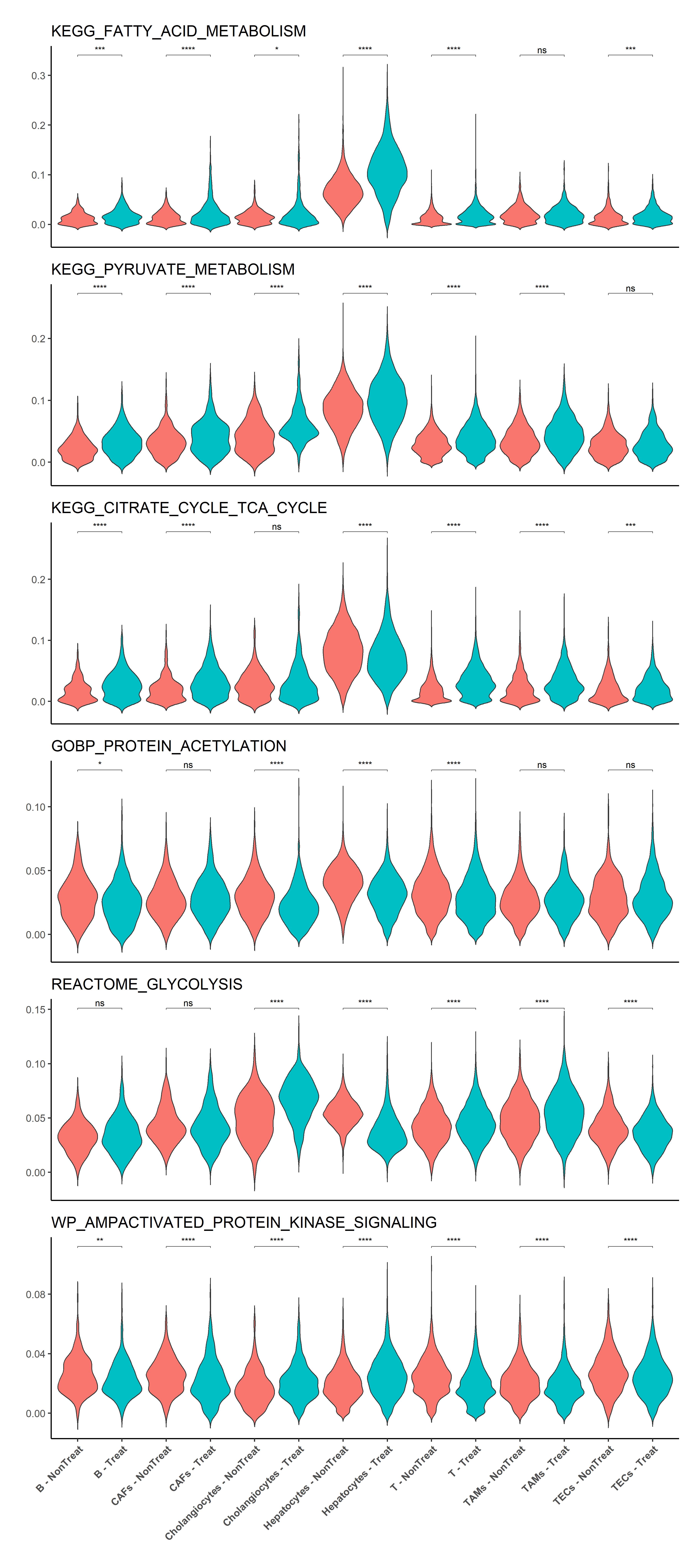

Supplement: Supplementary Figure 6 — Analysis of differences in pathway scores across different cell subpopulations before and after treatment. [file Image6.jpeg]

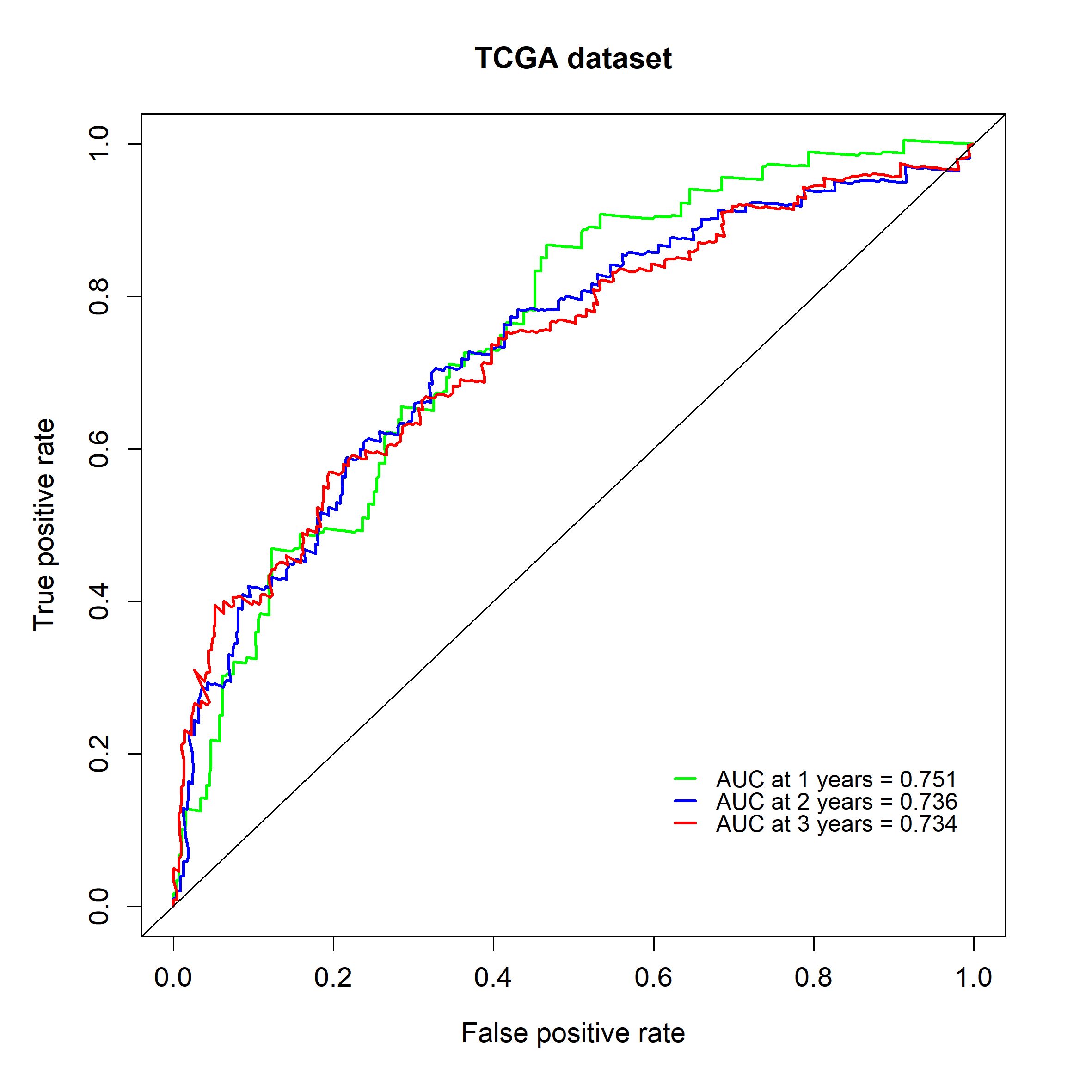

Supplement: Supplementary Figure 7 — The ROC curve is used to evaluate the reliability of the Lasso model, where an area under the curve (AUC) closer to 1.0 indicates better discriminative performance of the model for prognostic outcomes. [file Image7.jpeg]

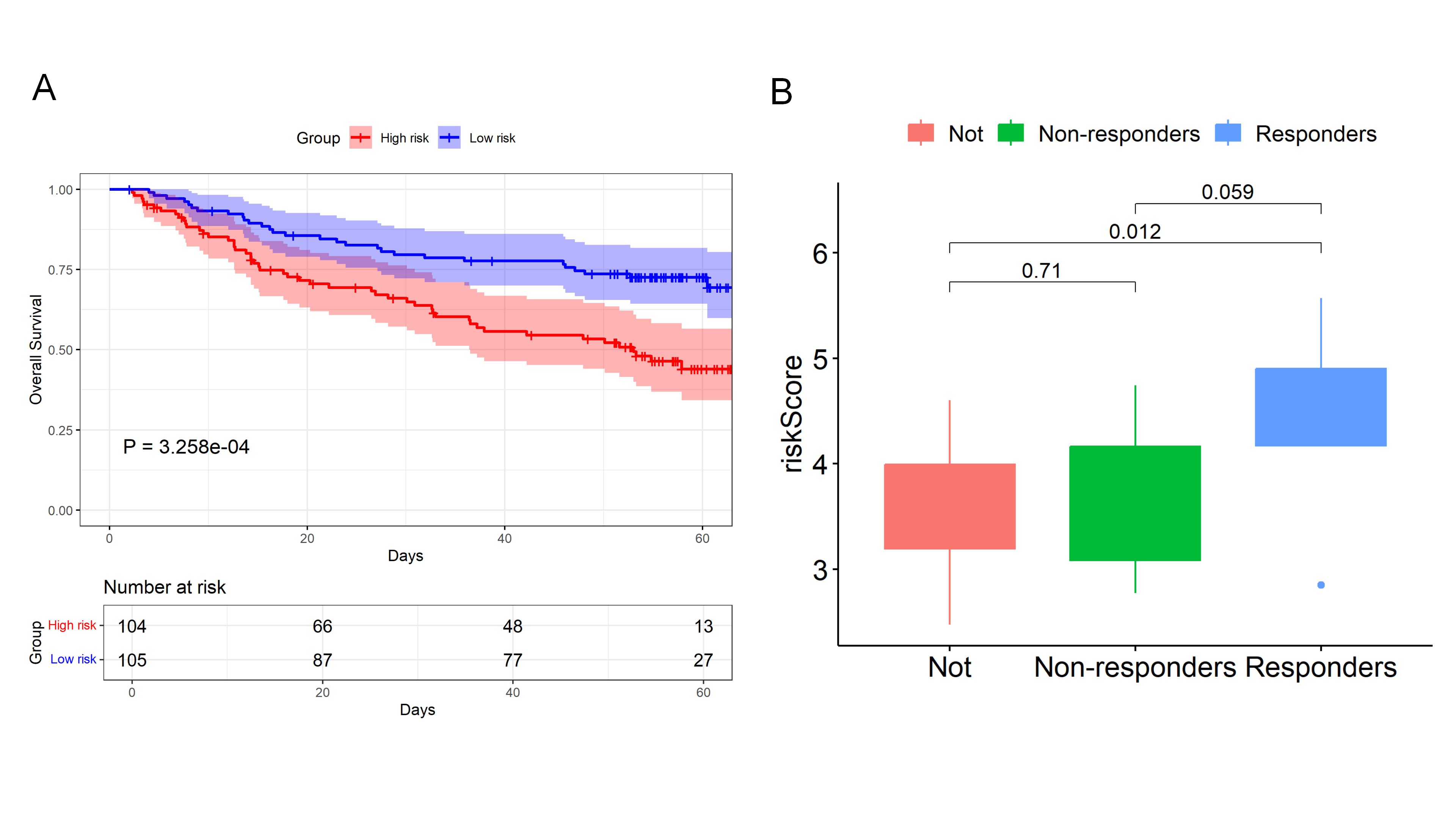

Supplement: Supplementary Figure 8 — External dataset validation. (A) The external dataset GSE14520 from the HCCDB database was incorporated to construct a prognostic model, and survival analysis was performed based on stratification into high- and low-risk score groups. (B) Bulk RNA-seq data of hepatocellular carcinoma before and after treatment were obtained, and differential analysis of risk scores was conducted among the untreated, treatment-nonresponsive, and treatment-responsive groups. A P value < 0.05 was considered statistically significant. [file Image8.jpeg]

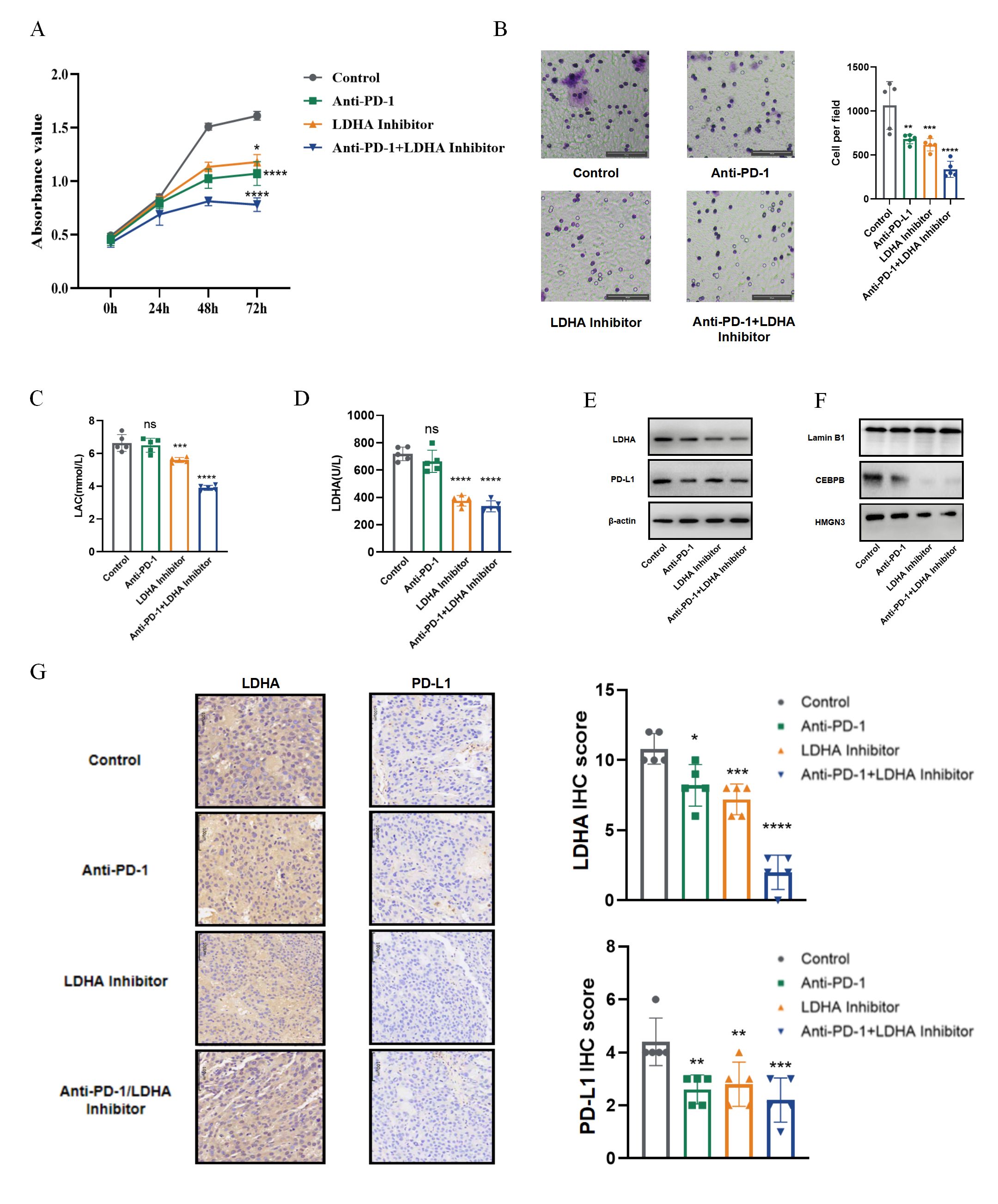

Supplement: Supplementary Figure 9 — In vitro and in vivo functional validation in HepG2 cells. (A) Cell viability of HepG2 cells in different treatment groups measured by CCK-8 assay. (B) Transwell migration assay evaluating the migratory capacity of HepG2 cells across different treatment groups. (C, D) Differential expression of LAC (C) and LDHA (D) among the treatment groups. (E) Western blot analysis of PD-L1 and LDHA protein expression in different treatment groups. (F) Western blot analysis of nuclear expression levels of HMGN3 and CEBPB in different treatment groups (Lamin B1 used as the nuclear loading control). (G) IHC staining of tumor tissues showing LDHA and PD-L1 expression across different treatment groups. Scale bar: 100μm. *p < 0.05; **p < 0.01; ***p < 0.001; ****p < 0.0001. [file Image9.jpeg]
